# Scalable synthesis and validation of PAMAM dendrimer‐*N*‐acetyl cysteine conjugate for potential translation

**DOI:** 10.1002/btm2.10094

**Published:** 2018-05-25

**Authors:** Rishi Sharma, Anjali Sharma, Siva P. Kambhampati, Rajsekar Rami Reddy, Zhi Zhang, Jeffrey L. Cleland, Sujatha Kannan, Rangaramanujam M. Kannan

**Affiliations:** ^1^ Center for Nanomedicine, Department of Ophthalmology Wilmer Eye Institute Johns Hopkins University School of Medicine Baltimore MD 21287; ^2^ Dept. of Anesthesiology and Critical Care Medicine Johns Hopkins University School of Medicine Baltimore MD 21287; ^3^ Ashvattha Therapeutics, LLC Baltimore MD 21218; ^4^ Hugo W. Moser Research Institute at Kennedy Krieger, Inc. Baltimore MD 21205; ^5^ Kennedy Krieger Institute – Johns Hopkins University for Cerebral Palsy Research Excellence Baltimore MD 21287; ^6^ Dept.of Chemical and Biomolecular Engineering Johns Hopkins University Baltimore MD 21218

**Keywords:** activated microglia targeting, antioxidant, dendrimer‐drug‐conjugate, *N*‐acetyl cysteine, neuroinflammation, PAMAM‐hydroxyl dendrimer, scalable synthesis

## Abstract

Dendrimer‐*N*‐acetyl cysteine (D‐NAC) conjugate has shown significant promise in multiple preclinical models of brain injury and is undergoing clinical translation. D‐NAC is a generation‐4 hydroxyl‐polyamidoamine dendrimer conjugate where *N*‐acetyl cysteine (NAC) is covalently bound through disulfide linkages on the surface of the dendrimer. It has shown remarkable potential to selectively target and deliver NAC to activated microglia and astrocytes at the site of brain injury in several animal models, producing remarkable improvements in neurological outcomes at a fraction of the free drug dose. Here we present a highly efficient, scalable, greener, well‐defined route to the synthesis of D‐NAC, and validate the structure, stability and activity to define the benchmarks for this compound. This newly developed synthetic route has significantly reduced the synthesis time from three weeks to one week, uses industry‐friendly solvents/reagents, and involves simple purification procedures, potentially enabling efficient scale up.

## 1 INTRODUCTION

Drug development for central nervous system (CNS) disorders is highly challenging as compared to other disease targets. The critical obstacle in this endeavor is the delivery of therapeutics across the blood brain barrier (BBB) which controls the passage of molecules from circulatory system into the brain.^1^, ^2^ Various invasive and non‐invasive techniques are currently being explored to get appropriate exposure of therapeutics into the brain.^1^, ^3^, ^4^, ^5^ Invasive strategies involve the temporary disruption of the BBB or direct injections into CNS tissues, while non‐invasive approaches primarily utilize endogenous cellular mechanisms for enhanced delivery across the BBB. These approaches can be valuable but may not be feasible in clinical settings when multiple therapeutic doses are required. In addition, the need to further deliver the drugs to cells far away from the BBB is a challenge. Neuroinflammation, mediated by activated microglia and astrocytes, has been shown to play a key role in various neurological diseases making it a potential therapeutic target.^6^, ^7^, ^8^, ^9^ Neuroinflammation causes transient disruption of the BBB through loosening of the tight junctions of the epithelial layer, resulting in increased permeability under certain pathological conditions.^10^ This impaired BBB in neuroinflammatory disorders presents an excellent opportunity to transport therapeutic molecules or drug‐loaded nanoparticles into the brain via intravenous administration.

Recently, the field of drug delivery has been revolutionized with a plethora of innovative nanotechnology‐based products which can effectively diffuse through the biological barricades within the body and deliver payloads of drugs, DNA, proteins or peptides to injured or diseased cells and tissues.^11^, ^12^, ^13^, ^14^, ^15^, ^16^ Despite the significant growth in the field of nanotechnology, nanoparticles face challenges during clinical translation. Major challenges include reproducible scale‐up of the synthesis, and definition of the benchmarks of the final product and the stability.^17^ Maintaining batch‐to‐batch consistency in physico‐chemical, pharmacological and biological properties is critical for enabling translation. The utility of a nano‐therapeutic in the clinic greatly depends upon its safety profile which is governed by its components, size, charge, and other physiochemical properties.^18^ The risk of failure can be minimized beforehand by carefully selecting rationally designed components that have already been tested and approved and can be incorporated together for the preparation of the particle.

Dendrimers are structurally “monodisperse” with well‐defined building blocks and surface functionality, and have garnered significant attention as promising scaffolds for various biomedical applications including drug/gene delivery, targeting, imaging and diagnosis due to their unique physico‐chemical properties.^19^, ^20^, ^21^, ^22^, ^23^, ^24^, ^25^ These multivalent nanostructures can be tuned conveniently to incorporate various molecules of interest. Amongst several different types of dendrimers, polyamidoamine (PAMAM) dendrimers have been most widely explored for drug delivery applications due to their commercial availability and aqueous solubility.^26^, ^27^, ^28^ We previously reported that non‐cytotoxic, hydroxyl terminated generation 4 PAMAM dendrimers (PAMAM‐G4‐OH, ∼4 nm size) can cross the impaired BBB upon systemic administration and target activated microglia and astrocytes at the site of injury in the brain several folds more than the healthy control in a neonatal brain injury model without any targeting ligand.^29^ We further validated these findings in various small and large animal models of neuroinflammtory disorders.^30^, ^31^, ^32^, ^33^, ^34^, ^35^, ^36^, ^37^ The selective uptake and localization of these neutral dendrimers in activated microglia might be attributed to their ability to cross the impaired BBB and diffuse rapidly within the brain parenchyma.^38^, ^39^ Due to their neutral surface, hydroxyl PAMAM dendrimers have a relatively low risk of opsonization upon systemic administration and exhibit low non‐specific interactions, which are a major drawback associated with cationic amine functionalized PAMAM dendrimers.^40^, ^41^, ^42^ Unlike their charged counterparts (PAMAM‐NH_2_ and PAMAM‐COOH), neutral PAMAM‐G4‐OH dendrimers are nontoxic even at intravenous doses greater than 500 mg/kg.^30^, ^41^

D‐NAC is a covalent conjugate of PAMAM‐G4‐OH attached to *N*‐acetyl cysteine (NAC) through a disulfide linker which can selectively release NAC intracellularly in the presence of glutathione.^30^ NAC is a clinically approved anti‐inflammatory and anti‐oxidative agent but needs to be given at high doses due to its poor bioavailability which can cause neuronal toxicity.^43^, ^44^, ^45^, ^46^ D‐NAC selectively delivers NAC to the inflamed areas of the brain, enhancing its efficacy, reducing collateral damage in healthy cells, and minimizing other off‐target effects. We have observed 100‐fold improved efficacy of D‐NAC when compared to equivalent doses of the free drug.^11^ D‐NAC has shown significant efficacy in a neonatal rabbit model of cerebral palsy,^30^ a mouse model of hypoxic‐ischemia,^31^ and other neuro‐inflammation models of various small and large animals when injected systemically.^32^, ^36^ Although we have successfully synthesized numerous batches of D‐NAC at the lab scale, in order to support our preclinical and clinical demands, we needed a highly optimized, well‐established, reproducible, reliable, and robust synthetic protocol which can lead to kilogram scale quantities with a high purity and quantitative yields in few reaction steps. Based on the critical analysis of our prior reported procedure, we here report a scalable improvised process for D‐NAC synthesis which can be transferred for cGMP manufacturing. The synthetic protocol discussed here for the construction of D‐NAC is highly robust and reproducible, involves industrial friendly solvents, is environmentally friendly, and provides rapid access to the final conjugate with ease. We have also evaluated the reproducibility of D‐NAC developed by this synthetic route by generating several batches with a similar drug payload. In addition, we developed another synthetic methodology to construct D‐NAC with modifications to the linker on the dendrimer. The resultant compound has been extensively characterized for structure, drug payload, stability and activity.

## 2 MATERIALS AND METHODS

### 2.1 Synthesis of intermediates and dendrimers

The detailed synthetic procedures are described in the Supporting Information.

### 2.2 Characterization

#### 2.2.1 Nuclear magnetic resonance

Nuclear magnetic resonance (NMR) spectra were recorded on a Bruker 500 MHz spectrometer at ambient temperatures. Proton and carbon chemical shifts (δ) are reported in ppm. The resonance multiplicity in the ^1^H NMR spectra are described as “s” (singlet), “d” (doublet), “t” (triplet), and “m” (multiplet) and broad resonances are indicated by “b”. Residual protic solvent of CDCl_3_ (^1^H, δ 7.27 ppm; 13C, δ 77.0 ppm (central resonance of the triplet)), DMSO‐*d*_6_ (^1^H, δ 2.50 ppm), and MeOD (^1^H, δ 3.31 ppm and 13C, δ 49.0 ppm) were used for chemical shifts calibration.

#### 2.2.2 Mass spectroscopy

Accurate mass measurements (HRMS) were performed on Bruker microTOF‐II mass spectrometer using ESI in positive mode and direct flow sample introduction in CH_3_CN:H_2_O (9:1) solvent system. Either protonated molecular ions [M + nH]^n+^ or adducts [M + nX]^n+^ (X = Na, K, NH_4_) were used for empirical formula confirmation. MALDI‐TOF experiments were performed on Bruker Autoflex MALDI‐TOF instrument. The conjugate was dissolved in Ultra purified water at 5 mg/mL and 2, 5‐dihydroxybenzoic acid (DHB) matrix was dissolved in 50:50 (v/v) acetonitrile:water mixture at 10 mg/mL concentration. The samples were prepared by mixing 10 µL of conjugate solution with 10 µL of DHB solution and 3 µL of the sample was spotted on a MALDI plate. Laser power used for this purpose was 55–100%

#### 2.2.3 High‐performance liquid chromatography

The purity of intermediates and final dendrimer conjugate were analyzed using high‐performance liquid chromatography (HPLC). HPLC (Waters Corporation, Milford, MA) is equipped with a 1525 binary pump, an In‐Line degasser AF, a 717 plus autosampler, a 2998 photodiode array detector, a 2475 multi *λ* fluorescence detector interfaced with Waters Empower software, and column used is symmetry 300 C18 5 µm, 4.6 × 250 mm. The HPLC chromatogram of the starting dendrimer (PAMAM‐G4‐OH), intermediates and D‐NAC were monitored at 210 nm. The Cy5‐D‐NAC was monitored at both 650 and 210 nm using PDA and fluorescence detectors. For PAMAM‐G4‐OH, intermediates and final conjugate, a gradient flow was used in HPLC starting with 100:0 (*Solvent A*: 0.1% TFA in 5% Acetonitrile (ACN) in water; *Solvent B*: 0.1% TFA in ACN); gradually increasing to 50:50 (A:B) at 20 min, returning to 90:10 at 30 min and finally to 100:0 at 40 min maintaining a flow rate of 1 mL/min. This HPLC method was used for stability study of D‐NAC and TCEP reduction. For Cy5 labeled dendrimer, and for all other HPLC analyses discussed in this paper, a similar method was used except the solvent A was 100% water instead of 5% ACN in water.

#### 2.2.4 Dynamic light scattering and zeta potential (ζ)

The particle size and ζ‐potential of D‐NAC were determined by using a Zetasizer Nano ZS (Malvern Instrument Ltd., Worchester, UK) equipped with a 50 mW He–Ne laser (633 nm). For Dynamic light scattering (DLS) measurement, the conjugate was dissolved in deionized water (18.2 Ω) to make solution with a final concentration of 0.5mg/mL. The OP‐101 sample solution for DLS is prepared fresh prior taking the measurement and the solution was vortexed for 1 min and then sonicated for 3 min. The solution was then filtered through 0.2 µm syringe filters (Pall Corporation, 0.2 µm HT Tuffryn membrane) directly into the cell (UV transparent disposable cuvette, Dimensions: 12.5 × 12.5 × 45mm, SARSTEDT). Specifications for the instrument: Material RI: 1.56, Material absorption: 0.001, Dispersant RI: 1.33, Viscosity: 0.8872 cP, Measurement position: automatic, Cell: DTS‐0012, Measurement angle: 173° Backscatter (NIBS default), Measurement type: Manual, Number of measurements: 3, Number of runs per measurement: 11, Run duration: 10 s. DLS measurements were performed in triplicate.

For zeta potential measurement, the ideal concentration for the measurement is 0.2 mg/mL in 10 mM NaCl. The pH of the solution is 5.6. The solution was filtered through 0.2 µm syringe filters (Pall Corporation, 0.2 µm HT Tuffryn membrane) directly into the cell (Malvern Zetasizer Nanoseries disposable folded capillary cells) and three measurements were taken. Specifications: Material RI: 1.56, Material absorption: 0.001, Dispersant RI: 1.330, Viscosity: 0.8894 cP, Measurement type: Manual, Number of measurements: 3, Number of runs per measurement: 20.

#### 2.2.5 Calculation of NAC loading by TCEP reduction protocol

The release of free NAC from dendrimer was achieved by the reduction with tris(2‐carboxy ethyl) phosphine hydrochloride (TCEP). D‐NAC (1 mg in HPLC grade water (600 µL) was incubated with TCEP (780 µg) in 400 µL HPLC grade water for 2 h at room temperature with constant stirring in a glass vial. The overall concentration of D‐NAC was 1 mg/mL. A calibration curve of free NAC was made using area under the curves (AUC) obtained from HPLC vs. amount, by injecting different samples of NAC ranging from 1 to 100 µg in HPLC. Hundred micro liters of sample solution from the incubation vial was injected to HPLC and AUC was recorded for NAC peak at retention time (∼5 min). The NAC content was calculated from the calibration curve.

### 2.3 In vitro efficacy evaluation of D‐NAC conjugates

#### 2.3.1 Cell culture

Murine brain microglia (BV‐2) passage 21 were cultured in phenol red free Dulbecco's modified Eagle's medium (DMEM; Life technologies, Grand Island, NY) supplemented with 5% heat in activated fetal bovine serum (Hi‐FBS; Invitrogen Corp., Carlsbad, CA) and 1% antibiotics (penicillin/streptomycin) (Invitrogen Corp., Carlsbad, CA). The cells were maintained in a humidified incubator at 37°C with 5% CO_2_.

#### 2.3.2 Cell toxicity and lactate dehydrogenase activity

BV‐2 cells were plated at a concentration of 10,000 cells/well in a 96 well plate (Costar, Cambridge, MA) and incubated at 37°C for 24 h allowing them to be attached to the well surface. After 24 h, the cells were treated with medium containing different concentrations (1–1,000 µg) of NAC, three batches of D‐NAC (containing equivalent amount of NAC in conjugated form) and G4‐OH (equivalent amount as that of D‐NAC) for 6 h. After 8 h the treatment medium was replaced with fresh medium for another 18 h. The cells treated with medium alone served as control. The cell viability was assessed using MTT cell proliferation assay kit (Molecular probes; Invitrogen, OR). Absorbance was read at 540 nm using fluorescence microplate reader (BioTek Instruments; Winooski, VT) and cell viability was determined as percent absorbance relative to untreated control cells. The LDH release mediated cell cytotoxicity by the NAC, D‐NAC (three batches) and G4‐OH treated cells were assessed using Pierce LDH cytotoxicity assay kit (Thermo Scientific, Waltham, MA). After treating the cells with NAC, D‐NAC and G4‐OH for 6 h, the treatment medium is replaced with fresh medium and the LDH medicated cytotoxicity was measured by following the kit manufacturer's instructions. The cells treated with medium alone served as spontaneous LDH activity controls and the cells treated with 10X lysis buffer (provided by the kit) served as maximum LDH release controls. The endpoint absorbance at 490 nm was measured using the plate reader.

#### 2.3.3 Anti‐inflammatory and anti‐oxidant activity of D‐NAC (3 batches) in microglial cells

Anti‐inflammatory and anti‐oxidant activity of D‐NAC was evaluated in BV‐2 cells using LPS activation as reported in previous established protocols.^47^, ^48^ BV‐2 cells were seeded in 12 well plates at a concentration of 1 × 10^6^ cells per well. The cells were pretreated with 100 ng/mL LPS (from *E. coli* 0127:B8, Lot#125M4091V) (Sigma‐Aldrich, St Louis, MO) for 3 h. After 3 h of LPS pre‐activation, the cells were treated with different concentrations (200, 100, 10 and 1 μg/mL) of NAC and three consecutive batches of D‐NAC (containing equivalent amount of NAC in conjugated form) for 6 h. After 6 h, the treatment medium was removed, and the cells were washed gently with warm PBS and replaced with culture medium containing LPS (100 ng/mL) for another 24 h. At the 24 h end‐point, the medium from the respective treated cells were collected and centrifuged at 5,000 rpm for 5 min at 4°C and immediately stored in −80°C until analysis. The anti‐inflammatory activity was accessed by measuring the levels of proinflammatory cytokine tumor necrosis factor‐α (TNF‐α) secreted by the cells in to the media. A mouse TNF‐α ELISA kit (Biolegend, San Diego, CA) was used and TNF‐α levels were measured by following the kit protocol. The end point was measured by measuring the absorbance at 450 nm with a wavelength correction at 570 nm. The media samples form cells with no LPS pre‐treatment (resting) and LPS treatment alone serves as controls. Anti‐oxidant activity of D‐NAC treatment on LPS activated BV‐2 cells was evaluated by measuring the nitric oxide (NO) released in the medium using a Greisess reagent detection kit (Promega, Madison, WI) by following the kit protocol. Briefly, the media samples were diluted appropriately, and the samples (50 μL each) were placed in 96‐well glass bottom, black‐walled plates (Corning, NY) and reacted with sulfanilamide and NED solutions provided in the kit. The end‐point of the reaction (color change, according to the kit's protocol) was measured using a plate reader at 520 nm with a correction at 550 nm. The NO levels were calculated using the calibration graph equation as per the manufacturer's instructions.

### 2.4 In vivo targeting of fluorescently labeled Cy5‐D‐NAC

#### 2.4.1 Rabbit model of CP and administration of D‐NAC‐Cy5

Timed‐pregnant New Zealand white rabbits were purchased from Robinson Services Inc. (North Carolina) and arrived at the facility one week before surgery. All animals were housed under ambient conditions (22°C, 50% relative humidity, and a 12‐h light/dark cycle), and necessary precautions were undertaken throughout the study to minimize pain and stress associated with the experimental treatments. Experimental procedures were approved by the Johns Hopkins University Animal Care and Use Committee (IACUC). After one week of acclimation, the pregnant rabbits underwent laparotomy on gestational day 28 (G28) and received a total of 4,800 EU of LPS (*E. coli* serotype O127:B8, Sigma‐Aldrich, St. Louis, MO) injection along the wall of the uterus as previously described.^49^, ^50^, ^51^ The kits were induced on G30 with intravenous injection of Pitocin (0.2 unit/kg) and kept in incubators with the temperature of ∼32–35°C and relative humidity of ∼50–60%. On postnatal day (PND1), kits received systemic administration (i.v.) of 55mg/kg Cy5‐D‐NAC and sacrificed at 24 h post‐injection.

#### 2.4.2 Immunohistochemistry

Kits were anesthetized and transcardially perfused with PBS, followed by 10% formalin. The brains were removed and post‐fixed in 10% formalin overnight and cryoprotected in graded sucrose solutions. Coronal sections (30 µm, 1:6 series) were incubated with goat anti‐IBA1 (1:250; Abcam, MA) overnight at 4°C. Sections were subsequently washed and incubated with fluorescent secondary antibodies (1:250; Life Technologies, MA) for 2 h at room temperature. Next, the sections were incubated with DAPI (1:1,000, Invitrogen) for 15 min. After washing, the slides were dried and cover slipped with mounting medium (Dako, Carpinteria, CA). Confocal images were acquired with a Zeiss ZEN LSM 710 (Zeiss, CA) and processed with ZEN software.

## 3 RESULTS AND DISCUSSION

**Figure 1:**
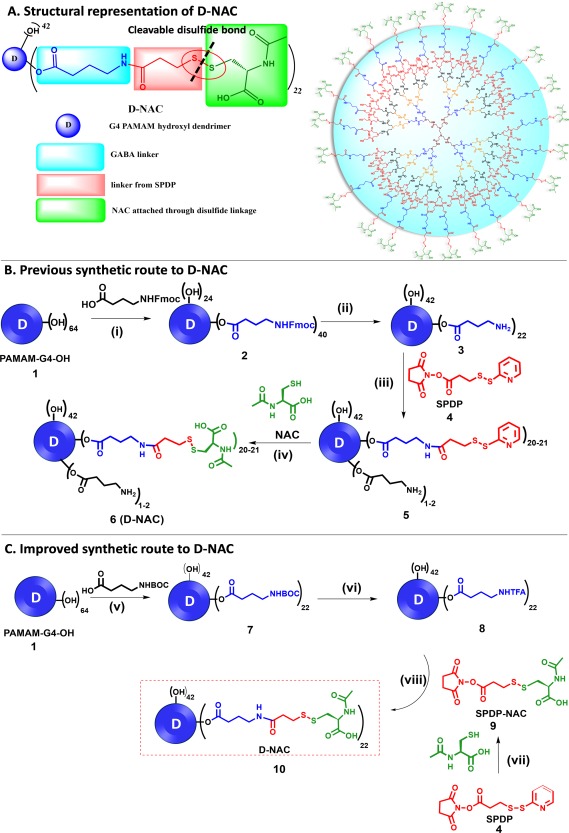
(A) Structural representation of D‐NAC demonstrating the individual components. (B) Previous synthetic pathway of D‐NAC: Reagents and conditions (i) PyBOP, DIEA, DMF/DMSO, RT, 48 h; (ii) Piperidine:DMF (2:8), RT, 2 h; (iii) DMF, *N*, *N*‐diisopropylethylamine (DIPEA), RT, 8h (iv) DMSO, RT, O/N. C. Improved synthetic pathway of D‐NAC: Reagents and conditions: (v) EDC, DMAP, DMF, 36 h, RT, 85%; (vi) DCM:TFA (4:1), RT,12 h, quantitative yield; and (vii) *N*‐Acetyl‐l‐cysteine, anhydrous THF, 2h, 65%. (viii) *N*, *N*‐diisopropylethylamine, pH 7.5, DMF, RT, 24 h, 90%

D‐NAC conjugate is built on a PAMAM‐G4‐OH dendrimer platform composed of an ethylene diamine core, repetitive branching units of amidoamine interior, and 64 terminal hydroxyl groups. The components of D‐NAC are: PAMAM‐G4‐OH dendrimer, GABA linkers attached via ester bonds, succinimidyl 3‐(2‐pyridyldithio)propionate (SPDP) linkers connected through amide linkages, and NAC molecules attached through glutathione‐sensitive disulfide bonds. D‐NAC contains an average of 22 ± 3 NAC molecules (Figure 1A). The starting dendrimer PAMAM‐G4‐OH (Pharma grade) is provided by Dendritech with more than 95% generational purity and >99% compositional purity. Dendritech has the potential to provide PAMAM‐G4‐OH at kilogram scale without compromising the purity levels. In order to meet our demand of D‐NAC for the eventual clinical studies, we need a well‐established, highly reproducible and robust synthetic methodology to construct this conjugate at kilogram scale. Here, we discuss the advantages of the improved synthesis over the preliminary protocol (Figure 1B,C). The improvised methodology was validated with multiple 10 g scale batches by our lab.

### 3.1 Synthesis of D‐NAC and advantages of improved protocol over the previous route

The previous protocol for D‐NAC production consisted of four synthetic steps (Figure 1B).^32^ The first step was the ligation of 4‐(fmoc‐amino)butyric acid with PAMAM‐G4‐OH (**1**) in the presence of PyBOP (benzotriazol‐1‐yl‐oxytripyrrolidinophosphonium hexafluorophosphate) as amide coupling reagent to obtain Fmoc‐protected bifunctional dendrimer (**2**), which was followed by the deprotection of Fmoc using piperidine (20% in DMF) to get bifunctional dendrimer (**3**). The amine termini of dendrimer (**3**) were then reacted with SPDP (**4**) to obtain pyridyldithio‐functionalized dendrimer (**5**) as an intermediate which was reacted *in situ* with NAC to yield D‐NAC (**6**). PAMAM‐G4‐OH is the most expensive material in the entire synthetic process. The choke point in the previous route was that all the reactions in the protocol were performed on the dendrimer. After each synthetic step on the dendrimer, the product was purified via elaborative dialysis in DMF for 24 h to remove small molecule impurities followed by another 24 h of water dialysis to eliminate DMF. We observed appreciable loss of dendrimer using this purification procedure. Reducing the number of reaction steps on the dendrimer can not only decrease the overall cost but also reduces the synthesis time. Moreover, we realized that since all of the synthesis steps were in sequence on the dendrimer in the previous protocol, it required at least 5–6 days (24 h reaction time, 48 h dialysis time, and 48 h lyophilization) to get the product from one reaction to use it as the reactant in the next step which could be troublesome for its large‐scale manufacturing in terms of time and cost. These issues will be critically evaluated one by one as we discuss the advantages of improved protocol.

To improve the scalability of the protocol, we divided the entire D‐NAC synthesis process into two halves. We elected to commence the synthesis in a semi‐convergent fashion by the construction of two main intermediates: (a) bifunctional dendrimer (**8**, Figure 1C) and (B) NAC‐SPDP (*N*‐acetyl‐S‐((3‐((2,5‐dioxopyrrolidin‐1‐yl)oxy)‐3‐oxopropyl)thio)cysteine) linker (**9**, Figure 1C). The final step involves the coupling of these two intermediates to yield the conjugate (D‐NAC, **10**). The major advantage of using this approach is that the synthesis of both the reactive partners can be started simultaneously which can drastically shorten the overall time frame of the synthesis. One of the most crucial steps for the preparation of the D‐NAC conjugate is the synthesis of bifunctional dendrimer having 22 ± 3 NH_2_ groups, as this is the step which determines the final NAC loading. In the previous synthesis, we used an Fmoc‐protected GABA linker. Although 20% piperidine/DMF is a widely used and universally accepted protocol for the removal of Fmoc from primary amines, in the case of Fmoc‐protected bifunctional dendrimer (**2,** Figure 1B), the ester linkage is prone to base‐catalyzed hydrolysis. The cocktail of piperidine:DMF used for the deprotection of Fmoc causes the partial cleavage of the linker from the dendrimer as well (40 NHFmoc in compound **2** to 22 free amines in compound **3**, Scheme 1A) consequently leading to inconsistency in the final loading of NAC in different batches. We first tried to fix this problem by optimizing the DMF:piperidine ratio by lowering the percentage of piperidine to avoid the cleavage of ester bonds but were not able to achieve complete deprotection of Fmoc. Additionally, piperidine is included in the list of controlled substances in the United States as it is precursor of psychoactive drugs and its use in industrial scale processes is not feasible due to administrative restrictions. To devise a suitably safe, reproducible, and scalable synthesis process, we sought to replace 4‐(fmoc‐amino)butyric acid in the reaction with BOC‐GABA‐OH.

PAMAM‐G4‐OH is received in methanol solution, which is evaporated completely using a rotary evaporator, followed by the dissolution in water and lyophilization. It is very important to remove the traces of methanol and water completely as these interfere in the coupling reaction. We then reacted PAMAM‐G4‐OH (**1,** Figure 1C) with BOC‐GABA‐OH in the presence of coupling agents [1‐Ethyl‐3‐(3‐dimethylaminopropyl)carbodiimide (EDC) and 4‐dimethylaminopyridine (DMAP)] in DMF for 36h at room temperature to yield BOC‐protected bifunctional dendrimer (**7**). The exact stoichiometry is described in the supporting information. The order of addition of reagents in the reaction should be followed exactly to obtain uniform product distribution with the desired loading of GABA‐BOC. The dendrimer, BOC‐GABA‐OH and DMAP are first dissolved in DMF under Argon atmosphere, followed by the addition of EDC.HCl in portions at the end. The progress of the reaction is monitored by HPLC to ensure the formation of product with the desired loading by comparing it with reference material. Upon completion, the precipitation of the crude product is achieved by adding an excess of hexanes, which is then decanted off. The precipitates are further washed with ethyl acetate to remove excess reagents and side products. The residue is then diluted with water and dialyzed using a 2 kDa membrane against ultra‐pure water for 24 h to further remove trace impurities. We observed that the diluted reaction mixture (30 mL water for 1 g of conjugate) provided the best yield during the dialysis process. In this step, we do not perform any DMF dialysis as all the reagents and side‐products are water‐soluble, which was not the case in previous protocol using Fmoc‐based reagents. The lyophilized product is then characterized by NMR and HPLC as further discussed in the characterization section. The BOC‐protected bifunctional dendrimer (**7**) is water soluble. If the bifunctional dendrimer at this stage is water insoluble, that is an indication of high loading of GABA BOC.

The next step involves the deprotection of BOC to obtain bifunctional dendrimer **8,** which is another critical phase of the synthesis. Fmoc deprotection step was the most problematic step in the previous synthesis, leading to inconsistencies in the overall drug loading. The BOC groups are conveniently deprotected under mild acidic conditions using trifluoroacetic acid (TFA) in DCM. It is critical to pay attention to the DCM:TFA ratio to produce desired results. The optimized ratio for the complete deprotection of BOC groups is 4:1. Since BOC‐protected dendrimer is partially soluble in the DCM:TFA mixture, the reaction results in a viscous mass which requires vigorous stirring for the effective completion of the reaction. After the deprotection, there is a considerable increase in the mass of the final product, which is due to the formation of TFA salts of the internal and external amines of the dendrimer. We consider the yield of this step 100% for the next chemical transformation. The replacement of BOC with Fmoc solved several problems initially encountered: (a) BOC deprotection is robust leading to reproducible results with no ester hydrolysis observed, (b) no further purification by dialysis is required, (c) excess TFA can be easily removed by co‐evaporation with methanol, and (d) no yield and time loss.

From the bifunctional dendrimer stage to the final D‐NAC conjugate, the previous protocol involved a two‐step reaction: addition of SPDP linker followed by a classical thiol‐disulfide interchange by NAC, dislodging the chromophoric pyridine‐2‐thione group, which took around a week including dialysis and lyophilization. Thiol‐exchange reaction is a widely used reaction for crosslinking and conjugation of molecules where a nucleophillic thiol participates in thiol‐disulfide interchange to form a new disulfide linkage. Although thiol‐exchange reaction is moderately efficient, replacing 22 ± 3 pyridine‐2‐thione (PDT) with NAC on the surface of dendrimer is challenging and often leads to incomplete reactions, leaving a few groups of PDT intact as determined by NMR, giving a yellow color to the final product and inconsistent NAC loading. In the newly developed protocol, we separately synthesized NAC‐SPDP (*N*‐acetyl‐S‐((3‐((2,5‐dioxopyrrolidin‐1‐yl)oxy)‐3‐oxopropyl)thio)cysteine) by reacting NAC with SPDP in tetrahydrofuran. The reaction was monitored by thin layer chromatography (TLC). If the reaction does not go to completion in 4 h, it can be pushed forward with the additional NAC. NAC should not be added in a huge excess as it can cause purification problems due to the close retention factor of the product on TLC. Since this disulfide exchange reaction is being carried out on a small molecule, it is highly efficient, and the yellow colored PDT side product is easily removed by column chromatography to obtain NAC‐SPDP (**9**) as a white crystalline solid with high purity. During the final ligation step, the TFA salt of bifunctional amine terminated dendrimer (**8**) is dissolved in DMF and neutralized using Hünig's base to get pH 7.0–7.5 followed by the slow addition of NAC‐SPDP (**9**) in DMF to yield D‐NAC (**10**). Implementation of these modifications produced D‐NAC as pure white solid. We have also found that using a large excess of NAC‐SPDP can cause the inclusion of additional arms on the hydroxyl groups of the dendrimer changing the final loading of NAC. To avoid this, an exact stoichiometry of NAC‐SPDP to the number of amine groups in the bifunctional dendrimer is used. Another precaution is to keep track of the pH during the reaction as we have noticed in large scale reactions there is slight drop in pH after the addition of SPDP‐NAC due to the presence of free carboxylic acid groups. This change in pH can be adjusted by additional Hünig's base. The pH should not exceed 7.5 to avoid the hydrolysis of the NHS ester of NAC‐SPDP.

In short, the improved protocol provides the following advantages: (a) shortens the over‐all synthesis time from three weeks to one week, (b) cuts down the production cost drastically, (c) provides the desired and reproducible narrow range of NAC loading, (d) produces a highly pure, white product, and (d) promotes a green synthesis using industry‐friendly reagents and solvents.

### 3.2 Characterization of intermediates and final D‐NAC conjugate

**Figure 2:**
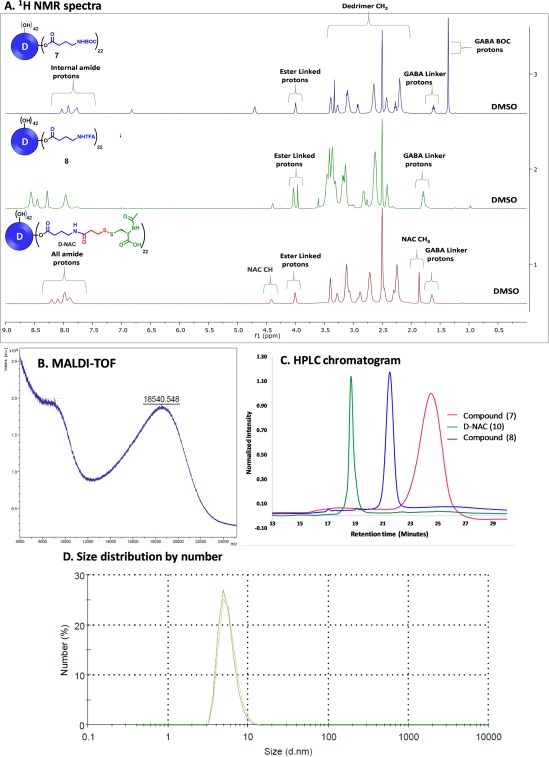
(A) ^1^H NMR comparison of intermediates and final D‐NAC conjugate, (B) MALDI‐TOF spectrum of the final conjugate, (C) HPLC comparison of the intermediates and final conjugate, and (D) size distribution by number for D‐NAC showing a size of 5.8 ± 0.2 nm

All of the intermediates and the final D‐NAC conjugate were characterized using NMR, HPLC, MALDI, or LCMS. Figure 2A depicts the proton NMR comparison of intermediates and the final conjugate from top to bottom. ^1^H NMR is one of the most robust and precise technique to characterize and calculate the exact loading of the conjugate. All of the dendrimers related NMRs are taken in DMSO‐*d*_6_ to see the internal amide protons of the dendrimer which are the reference protons to calculate the loading by NMR integration method. The ^1^H NMR of dendrimer **7** containing BOC‐protected GABA linkers clearly showed the presence of a sharp peak corresponding to *tert*‐butyl protons of BOC at δ 1.3 ppm along with GABA methylene protons at δ 1.6 ppm. A peak at δ 3.9 ppm is observed for the methylene protons of the dendrimer next to the hydroxyl groups once they are converted to ester. In addition, the amidic protons from the GABA linker can be seen at δ 6.8 ppm. For the exact calculation of the GABA loading, we compared the integration of the internal amide protons (124 H, δ 8–7 ppm) to the BOC protons, ester methylene protons and amide protons of the GABA linker. Another advantage of using BOC over Fmoc is the ease in calculating the exact loading of GABA linker by NMR. In case of Fmoc, the protons corresponding to the aromatic rings of Fmoc overlap with the internal amide protons of dendrimer causing inconvenience in calculation of exact loading of the linker, but in the case of GABA‐BOC, BOC protons do not interfere with any protons of the dendrimer and moreover, give a single sharp peak of 9 H corresponding to the *tert*‐butyl group for every unit of GABA attached, providing an accurate determination of the GABA loading (Figure 2A, top NMR). BOC deprotection is confirmed by the complete disappearance of the huge peak of BOC protons (Figure 2A, middle NMR). Finally, the attachment of NAC is confirmed by the appearance of *N*‐acetyl protons peak at δ 1.8 ppm and ‐C*H* of the NAC at δ 4.4 ppm (Figure 2A, bottom NMR). The NAC loading is calculated by comparing the GABA methylene protons and ester methylene protons to the *N*‐acetyl protons and ‐C*H* from the NAC molecules. The peak corresponding to the internal amide protons includes additional amidic protons from GABA linker and NAC. The average molecular weight of D‐NAC is analyzed using MALDI‐TOF analysis and fits well within the range corresponding to the attachment of 22 ± 3 NAC molecules (Figure 2B). The purity of D‐NAC is evaluated using HPLC (Figure 2C). The HPLC traces showed a clear shift in retention time at every stage. We have validated several batches of D‐NAC using this improved protocol with more than 98% purity. We further determined the size of D‐NAC to be around 5.8 nm using dynamic light scattering (Figure 2D, Table 1). Zeta potential measurement provided the value around 6.5 mV (Table 1).

**Table 1:** Size and zeta potential distribution of D‐NAC using dynamic light scattering

Sample	Theoretical M Wt. (g/mole)	Size (nm)	Zeta potential (ζ, mV)
**D‐NAC**	20,000 ± 2,000	5.8 ± 0.2	+ 6.5 ± 0.9
**PAMAM‐G4‐OH**	14,279	4.8 ± 0.3	+ 4.5 ± 0.6

### 3.3 Calculation of NAC loading by tris(2‐carboxy ethyl) phosphine hydrochloride reduction method

**Figure 3:**
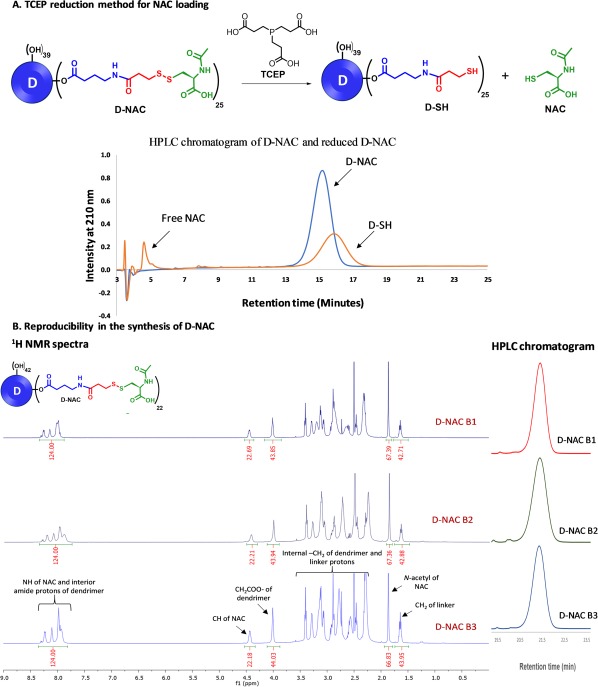
(A) Calculation of NAC loading by the TCEP reduction method (top: Reduction of D‐NAC by TCEP to produce free NAC and D‐SH, bottom: HPLC profile of D‐NAC and the reduced reaction mixture containing free NAC and D‐SH); (B) Confirmation of reproducibility of NAC loading (left: ^1^H NMR spectra of three different batches of D‐NAC representing reproducibility for consistent loading of NAC, right: HPLC chromatogram of different batches showing consistency in the purity of conjugate)

In addition to the NAC loading by proton integration method using ^1^H NMR, we further confirm NAC loading by the tris(2‐carboxy ethyl) phosphine hydrochloride (TCEP) reduction method (Figure 3A). We reduced the disulfide linkages in D‐NAC using an excess amount of TCEP to obtain free NAC and thiol‐terminating dendrimer (D‐SH). Compared with other disulfide reduction methods, the advantages of using TCEP are that it is: (a) a powerful reducing agent, (b) odorless, (c) resistant to air oxidation and (d) non‐interfering with the thiol containing species formed. The completion of the reaction is monitored by HPLC. The reduced dendrimer shows a shift in retention time from 14.93 min (D‐NAC) to 17.79 min (D‐SH). Once disulfide bonds are cleaved, the peak corresponding to free NAC at 5.18 min appears in the HPLC chromatogram. The amount of free NAC is calculated using HPLC and comparing the area under the curve with a standard calibration curve of NAC. Like the proton integration method for calculation of NAC loading, the TCEP reduction method was found to be highly precise and clean, producing similar results (22 ± 3 NAC molecules per dendrimer).

### 3.4 Reproducibility of D‐NAC synthesis

The most important aim of the improved synthetic protocol is to get a reproducible D‐NAC product at the end of synthesis with consistent NAC loading between batches of different scales. To validate the reproducibility of the improved method, we constructed several batches of D‐NAC and compared their ^1^H NMRs, NAC loading, and purity by HPLC. ^1^H NMR spectra of three different batches (Figure 3B left, Table 2) depict the presence of around 22 ± 3 NAC molecules, showing consistency in the synthesis of D‐NAC. HPLC chromatogram (Figure 3B right, Table 2) of these three batches of D‐NAC show the corresponding peaks at same retention time, confirming the consistency of NAC loading and reproducibility of the improved synthetic protocol.

**Table 2:** Validation of reproducibility of different batches of D‐NAC

D‐NAC	Batch 1	Batch 2	Batch 3	Average
NAC payload (Number of NAC molecules per dendrimer)	22	23	22	22
HPLC purity	99.8%	99.8%	99.7%	99.8%
HPLC retention time	16.3 min	16.2 min	15.9 min	16.1 min
Solubility	>200 mg/mL	>200 mg/mL	>200 mg/mL	>200 mg/mL
% Cell viability at 1,000 µg/mL	>90%	>90%	>90%	>90%
LDH‐mediated cytotoxicity at 1,000 µg/mL	∼10%	∼9.2%	∼7%	∼8.7%
Anti‐inflammatory activity (TNF‐α suppression at 100 µg/mL compared to free NAC)	∼2.1 fold	∼2.5 fold	∼2.7 fold	∼2.4 fold
Antioxidant activity (NO suppression at 100 µg/mL compared to free NAC)	∼1.5 fold	∼2.2 fold	∼2 fold	∼2 fold

### 3.5 Stability of D‐NAC formulation over time

**Figure 4:**
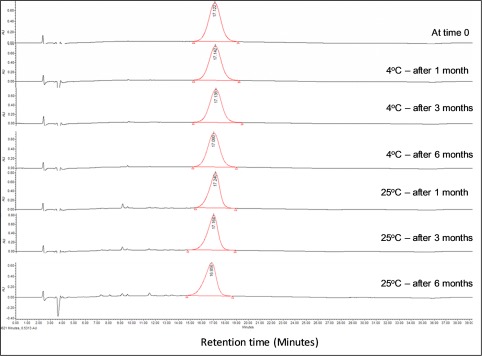
HPLC chromatogram demonstrating the stability of the D‐NAC formulation (200mg/mL) in 0.9% sterile saline over a period of 6 months at room temperature (25°C) and at 4°C

The stability of D‐NAC at physiological pH and *in vitro* release of NAC in the presence of glutathione has previously been investigated by our group.^30^ For the clinical use of D‐NAC, we prefer the drug as a formulation in 0.9% sterile and non‐pyrogenic saline solution with a concentration of 200 mg of conjugate per ml of solution. We further studied the stability of this D‐NAC formulation to evaluate its shelf life. The D‐NAC formulation was prepared by weighing powder form D‐NAC and then adding 0.9% of sterile saline in a pre‐sterilized laminar flow hood to achieve the final 200 mg/ml concentration. The solution was thoroughly vortexed and sonicated to ensure the formation of a uniform solution with complete dissolution of D‐NAC, followed by filtration through a sterile filter (0.2 µm). The stability of the formulation was studied at 4°C and 25°C for 2.7 mL of solution in 5 mL vials in triplicate over a period of 6 months. The samples were studied by HPLC at different time points (0, 1 month, 3 months, and 6 months). Figure 4 displays the HPLC traces of the D‐NAC formulation samples at various time points at 4°C and 25°C. At time zero the purity of the D‐NAC is 99.8%. The D‐NAC formulation (200mg/mL) in saline showed excellent stability at 4°C, with 99.4% purity even after 6 months. Minor additional peaks were observed at later time points at 25°C, but the purity of D‐NAC was still greater than 95% without any shift in retention time as compared to the main peak retention time at time zero. Moreover, even after 6 months at both temperatures, we did not observe any NAC release. Based on these results, 4°C is considered the appropriate temperature to store the D‐NAC formulation with excellent stability.

### 3.6 D‐NAC did not induce cell cytotoxicity or LDH‐mediated apoptosis in microglial cells between batches synthesized by improved protocol

**Figure 5:**
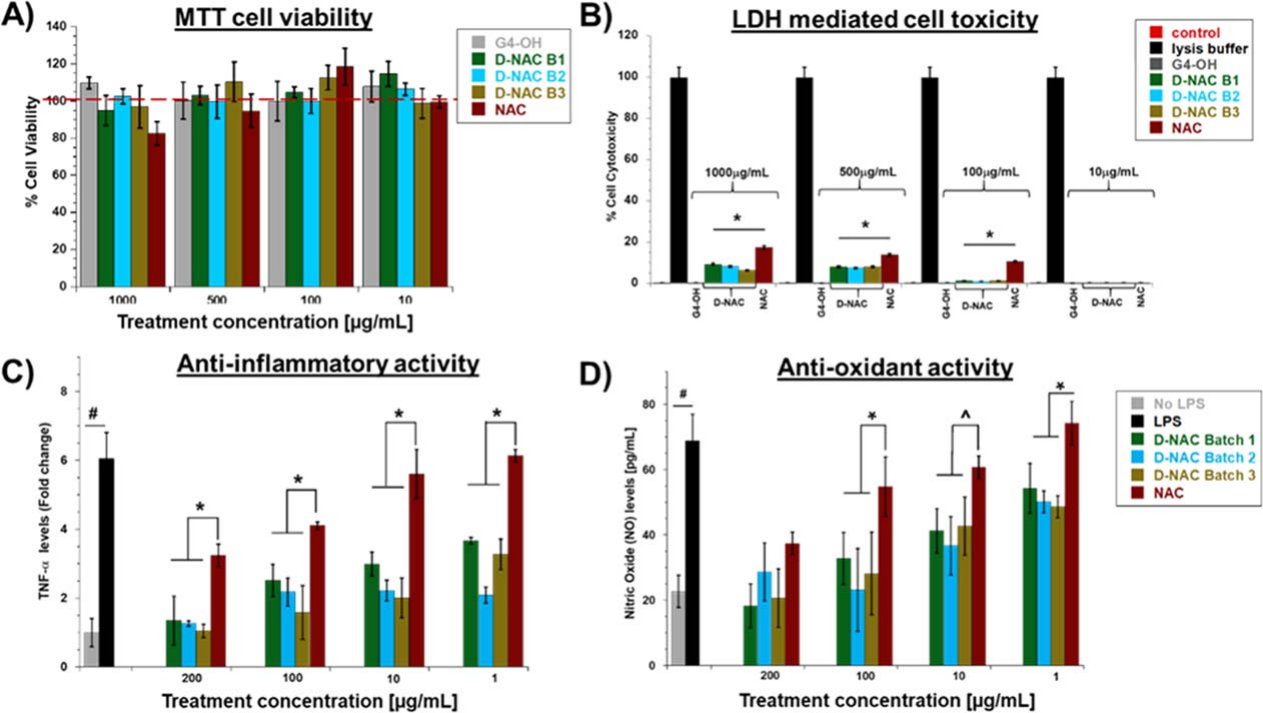
*In vitro* characterization of D‐NAC conjugates in murine microglial (BV‐2) cells. (A) Cytotoxicity profile of PAMAM G4‐OH, D‐NAC and NAC in BV‐2 cells upon 6 h treatment; (B) LDH mediated cytotoxicity showing minimal cells death due to apoptosis in D‐NAC treated groups compared to NAC (* *p* < .01, *n* = 6); (C) Anti‐inflammatory activity of D‐NAC in LPS activated microglial cells (# *t*‐test, *p* < .001, *n* = 4, * one‐way ANOVA, *p* < 0.001, *n* = 4); and (D) Anti‐oxidant activity of D‐NAC and NAC in LPS activated BV‐2 microglial cells (# *t*‐test, *p* < .001, *n* = 4, * one‐way ANOVA, *p* < 0.001, *n* = 4, ^ one way ANOVA, *p* <.01, *n* = 4)

Before proceeding to *in vitro* efficacy experiments, we evaluated the cell cytotoxicity of PAMAM‐G4‐OH, NAC and D‐NAC in BV‐2 cells to establish the therapeutic window. We have previously reported that D‐NAC does not induce any cytotoxicity to brain primary mixed glial cultures.^52^ In this study, we analyzed the results from three different batches of D‐NAC synthesized using new route and compared them for reproducibility (Figure 5A,B, Table 2). The cells were treated with NAC, D‐NAC and PAMAM‐G4‐OH for a brief period of 6 h to ensure maximum uptake^52^, ^53^ and to mimic the *in vivo* blood circulation of the conjugate. At concentrations of 500 and 1,000 μg/ml, both D‐NAC (three batches) and PAMAM‐G4‐OH did not induce cytotoxicity to the cells. The percent cell viability was greater than 98% for both D‐NAC and PAMAM‐G4‐OH suggesting that the intracellular dendrimers did not induce any cell death. Whereas free NAC treatment caused ∼20.0% cell death at 1,000 μg (Figure 5A). At concentrations below 500 μg, NAC did not show any signs of cell death if treated for 6 h. Next, we assessed if three different D‐NAC batches caused LDH release induced cytotoxicity. LDH is an enzyme released by the cells when they are undergoing apoptotic cycle due to stress caused by toxic compounds intracellularly.^54^ Thus, we used the LDH‐mediated cytotoxicity as a measure of cell toxicity caused by internalized NAC, D‐NAC or PAMAM‐G4‐OH by the cells. PAMAM‐G4‐OH treatment at all concentrations did not demonstrate any signs of cell death or LDH‐mediated cytotoxicity (∼100% of cells were viable). D‐NAC treatment at 1,000 and 500 μg demonstrated very minimal cytotoxicity (∼9.5 ± 1.1% and 8.0 ± 0.6%, respectively) (Figure 5B). We suspect that this minimal toxicity may be due to the conjugation of NAC on the dendrimer surface since free NAC at treatment concentrations of 100–1,000 μg also showed LDH mediated cytotoxicity of ∼14.4 ± 0.9%, 12.6 ± 0.5%, and 10.6 ± 0.7%, respectively (Figure 5B).

### 3.7 Enhanced and reproducible anti‐inflammatory and anti‐oxidant activity of D‐NAC in LPS‐induced microglial cells

For investigating the anti‐inflammatory activity of D‐NAC, we used LPS‐induced pro‐inflammatory BV‐2 cells as an *in vitro* model of inflammation. LPS induces the release of inflammatory cytokines by activating the protein kinases via toll like receptors (TLR) pathway. The levels of TNF‐α released following 6 h treatment of NAC and D‐NAC were assessed as a measure of anti‐inflammatory effect. Since the cells were treated NAC and D‐NAC (containing equivalent NAC in conjugated form) for 6 h followed by treatment with LPS containing medium, the results reflect the therapeutic activity of NAC or D‐NAC taken up by the cells for a limited period and under continuous LPS stimulation. Upon exposure of cells to LPS resulted in a ∼6‐fold increase in TNF‐α release by BV‐2 cells. At 100 and 200 μg/mL treatment concentration, both NAC and D‐NAC were effective in suppressing TNF‐α production. All the three batches of D‐NAC were significantly more effective (∼2.5‐fold, *p* < .001) than NAC (Figure 5C, Table 2). NAC at 1 and 10 μg/mL did not attenuate production of TNF‐α, whereas D‐NAC demonstrated significant suppression. Interestingly, the TNF‐α levels from the cells treated with 1μg/mL D‐NAC were similar to that of 100 μg/mL NAC (Figure 5C). There was no significant difference in TNF‐α suppression among the three batches at all the treatment concentrations demonstrates reproducible efficacy of D‐NAC. This is due to the fact that all the three batches have similar drug loading and purity. Anti‐oxidant activity was evaluated by measuring the nitric oxide (NO) levels released by the cells in to the medium using Griess reagent kit. Activated microglial cells (LPS exposed) showed significant NO release (∼3.0‐fold) compared to non‐LPS treated controls (Figure 5D, Table 2). D‐NAC treatment at 10, 100 and 200 μg/mL concentrations significantly decreased NO production. In contrast, only 200 μg/mL dose of NAC was effective in decreasing the level of NO release (Figure 5D). This enhanced efficacy can be attributed to enhanced cellular uptake of dendrimers.^47^ We have previously demonstrated that the dendrimer uptake is increased when the microglial cells are activated.^55^ D‐NAC treatment improves intracellular glutathione (GSH) levels while suppressing glutamate release significantly compared to NAC.^52^

### 3.8 Fluorescently labeled D‐NAC localizes with activated microglia at white matter areas of CP rabbit kits

**Figure 6:**
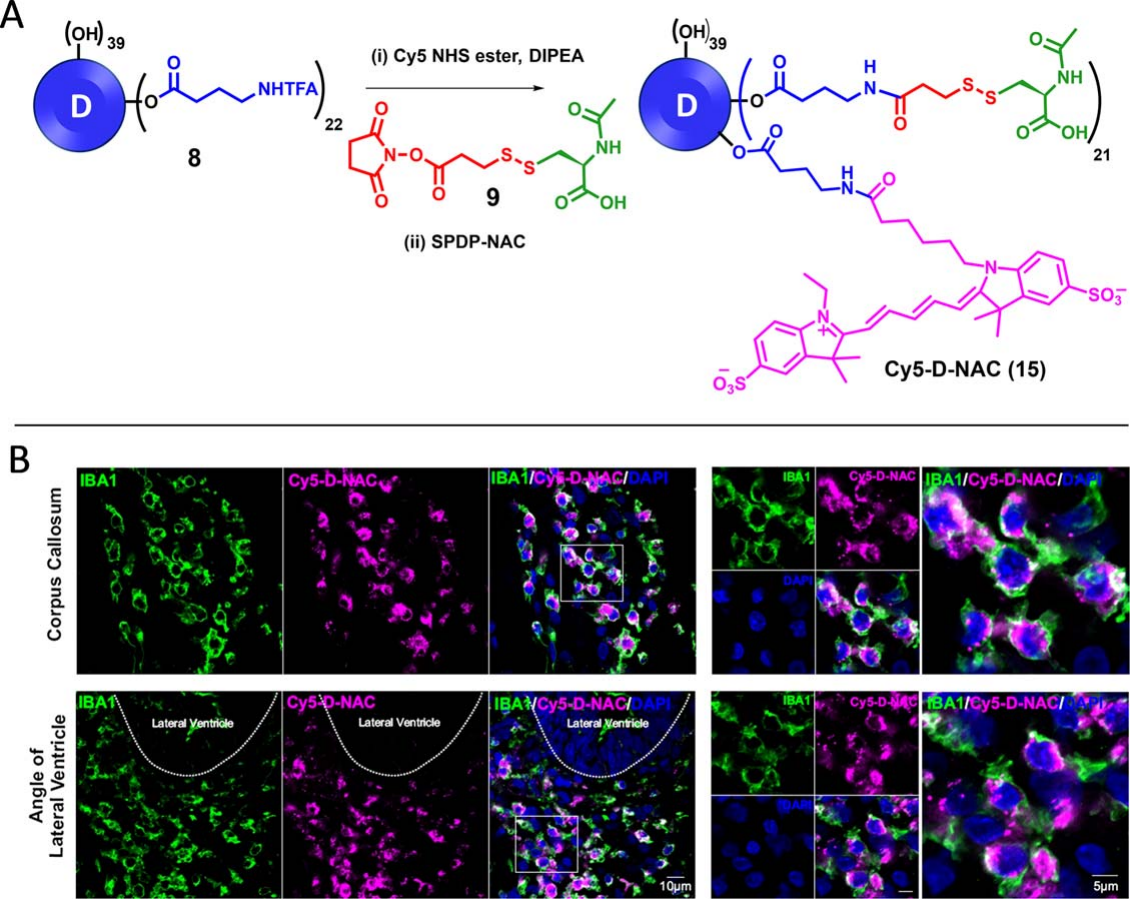
(A) Synthetic route to Cy5‐D‐NAC (15), (B) Cy5‐D‐NAC and microglial co‐localization in endotoxin kits. Brain slices were stained with IBA1 (microglial marker). Images were randomly acquired from the corpus callosum and lateral ventricle (white matter) of CP kits. Intense Cy5‐D‐NAC signal is co‐localized with activated microglia at both the corpus callosum and lateral ventricle in CP kits. Right panels are the higher magnification images indicated by the boxes on the images of the left panels. Scale bars: 10 μm (left panel) and 5 μm (right panel)

We conjugated a fluorescent tag cyanine 5 (Cy5) to D‐NAC to study brain uptake upon systemic administration in a neonatal rabbit model of CP with significant microglial activation.^30^ Figure 6A presents the synthesis route of Cy5‐D‐NAC (**15**). Bifunctional dendrimer (**8**) was first reacted with Cy5 NHS ester at pH 7.5 for 2 h followed by the addition of NAC‐SPDP (**9**) in the same pot. The product formation was confirmed with NMR and HPLC (Supporting Information).

The CP kits (*n* = 3) received an intravenous administration of Cy5‐D‐NAC on PND1 and were sacrificed 24 h post‐injection. We found that the Cy5‐D‐NAC conjugate co‐localized with activated microglia, indicated by ameboid soma with shortened processes, in the periventricular white matter region (PVR), including corpus callosum, and in the lateral ventricle in the cortex (Figure 6B). These results indicate that D‐NAC crossed the blood‐brain barrier, reached the injured white matter region, and targeted activated microglia in a cell‐specific manner consistent with our many previous findings.^30^, ^32^, ^52^ These properties make D‐NAC an ideal therapeutic reagent in the treatment of neonatal brain injuries.

### 3.9 Another synthetic approach for conjugation of NAC on dendrimer surface

**Figure 7:**
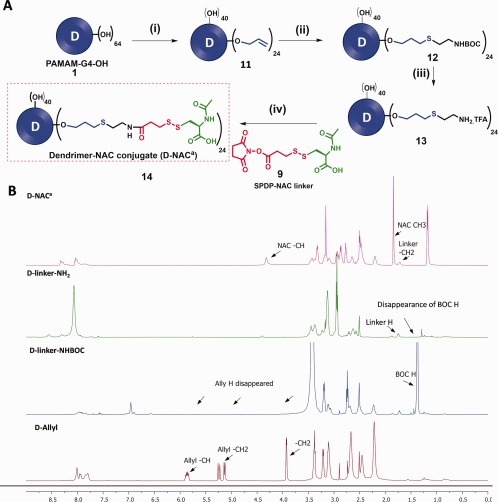
(A) Key steps in synthetic pathway of D‐NAC^a^ (14) Reagents and conditions: (i) Allyl bromide, NaH, DMF, 24 h, 45% (ii) BOC‐aminoethanethiol, DMPAP, UV, RT, 24 h,78%; (iii) DCM, TFA, RT, 24 h, quantitative yield; (iv) *N*, *N*‐diisopropylethylamine, pH 7.5, DMF, RT, 24 h, 62%; (B) ^1^H NMR comparison of intermediates and final dendrimer‐NAC conjugate via ether linker (D‐NAC^a^)

While we were redesigning the synthesis of D‐NAC, we also developed another synthetic route which can yield dendrimer‐NAC conjugate. In this route, we generated ether bound linker on the dendrimer surface. The ether linkages are robust, do not undergo hydrolysis and are not substrates of esterase. For this purpose, we reacted PAMAM‐G4‐OH (**1**) with allyl bromide in the presence of sodium hydride to conjugate 22–24 allyl arms on the dendrimer surface by ether bonds (**11**, Figure 7A). The alkene groups on dendrimer surface were then reacted with BOC‐aminoethane thiol under UV light (365 nm) using highly efficient and orthogonal photocatalyzed thiol‐ene click reaction to afford BOC protected dendrimer **12**. Thiol‐ene click is a highly robust and scalable reaction; and has been tremendously used to fabricate several dendrimer and polymer‐based macromolecular systems.^56^, ^57^ The completion of the reaction was confirmed by ^1^H NMR showing complete disappearance of allyl signals at δ 5.8, 5.2 and 3.9 ppm and appearance of a sharp peak for BOC protons at δ 1.3 ppm (Figure 7B). The BOC was then deprotected using TFA following similar conditions as described earlier to afford bifunctional ether linked dendrimer (**13**). The analysis of proton NMR clearly revealed the disappearance of *tert*‐butyl peak corresponding to BOC protons in the spectrum (Figure 7B). The bifunctional dendrimer conjugate **13** was finally reacted with SPDP‐NAC‐linker (**9**) to afford D‐NAC^a^ (**14**) with ether linkages on dendrimer surface. All the intermediates and final conjugate were characterized by ^1^H NMR and HPLC.

## 4 CONCLUSIONS

In summary, we presented a highly facile, convenient and robust protocol for the large‐scale preparation of D‐NAC which is superior to the previously adapted methods. The newly developed synthetic process produces a well‐defined D‐NAC in significantly shorter time‐frame, avoids the use of harsh chemicals and reagents, and has improved purification protocols which leads to the higher yields of product at every step involved. This protocol not only decreases the manufacturing cost drastically by cutting down the synthesis time but also trouble shoots the synthetic problems associated with the previous route; and provides a highly pure and monodispersed D‐NAC conjugate with high precision in different batches. The conjugates showed shelf‐stability over a 6‐month period when stored as a saline solution, and showed superior activity compared to free drug. Given the improved activity of polymer‐drug and dendrimer‐drug conjugates, this scale up protocol and characterization may offer insights into developing and defining such conjugates for eventual clinical translation.

## CONFLICT OF INTEREST

The authors declare no conflict of interest.

## Supporting information

Additional Supporting Information may be found online in the supporting information tab for this article.

Supporting Information

## References

[1] David SH , Aniket SW , Nathan BR , et al. Evolving drug delivery strategies to overcome the blood brain barrier. Curr Pharm Des. 2016;22(9):1177–1193. 26685681 10.2174/1381612822666151221150733 PMC4900538

[2] Krol S. Challenges in drug delivery to the brain: Nature is against us. J Control Release. 2012; 164(2):145–155. 22609350 10.1016/j.jconrel.2012.04.044

[3] Oberoi RK , Parrish KE , Sio TT , Mittapalli RK , Elmquist WF , Sarkaria JN. Strategies to improve delivery of anticancer drugs across the blood–brain barrier to treat glioblastoma. Neuro Oncol. 2016; 18(1):27–36. 26359209 10.1093/neuonc/nov164 PMC4677418

[4] Zhao G , Huang Q , Wang F , et al. Targeted shRNA‐loaded liposome complex combined with focused ultrasound for blood brain barrier disruption and suppressing glioma growth. Cancer Lett. 2018; 418:147–158. 29339208 10.1016/j.canlet.2018.01.035

[5] Fang F , Zou D , Wang W , et al. Non‐invasive approaches for drug delivery to the brain based on the receptor mediated transport. Mater. Sci. Eng. C 2017; 76:1316–1327. 10.1016/j.msec.2017.02.056 28482500

[6] Perry VH , Nicoll JAR , Holmes C. Microglia in neurodegenerative disease. Nat Rev Neurol. 2010;6(4):193 20234358 10.1038/nrneurol.2010.17

[7] Maragakis NJ , Rothstein JD. Mechanisms of disease: astrocytes in neurodegenerative disease. Nat Clin Pract Neurol. 2006;2(12):679 17117171 10.1038/ncpneuro0355

[8] Dommergues MA , Plaisant F , Verney C , Gressens P. Early microglial activation following neonatal excitotoxic brain damage in mice: a potential target for neuroprotection. Neuroscience 2003; 121(3):619–628. 14568022 10.1016/s0306-4522(03)00558-x

[9] Fuller S , Münch G , Steele M. Activated astrocytes: a therapeutic target in Alzheimer's disease? Expert Rev Neurother. 2009; 9(11):1585–1594. 19903019 10.1586/ern.09.111

[10] Varatharaj A , Galea I. The blood‐brain barrier in systemic inflammation. Brain Behav Immun. 2017; 60:1–12. 26995317 10.1016/j.bbi.2016.03.010

[11] Mann AP , Scodeller P , Hussain S , et al. A peptide for targeted, systemic delivery of imaging and therapeutic compounds into acute brain injuries. Nat Comms. 2016; 7:11980 10.1038/ncomms11980 PMC4931241 27351915

[12] Cui W , Li J , Decher G. Self‐Assembled Smart Nanocarriers for Targeted Drug Delivery. Adv Mater Weinheim. 2016; 28(6):1302–1311. 26436442 10.1002/adma.201502479

[13] Tsou Y‐H , Zhang X‐Q , Zhu H , Syed S , Xu X. Drug delivery to the brain across the blood–brain barrier using nanomaterials. Small 2017; 13(43):17019211701921‐n/a. 10.1002/smll.201701921 29045030

[14] Sharma A , Khatchadourian A , Khanna K , Sharma R , Kakkar A , Maysinger D. Multivalent niacin nanoconjugates for delivery to cytoplasmic lipid droplets. Biomaterials 2011; 32(5):1419–1429. 21071082 10.1016/j.biomaterials.2010.10.025

[15] Sharma A , Mejia D , Maysinger D , Kakkar A. Design and synthesis of multifunctional traceable dendrimers for visualizing drug delivery. RSC Adv. 2014; 4(37):19242–19245.

[16] Sharma A , Neibert K , Sharma R , Hourani R , Maysinger D , Kakkar A. Facile construction of multifunctional nanocarriers using sequential click chemistry for applications in biology. Macromolecules 2011; 44(3):521–529.

[17] Wei A , Mehtala JG , Patri AK. Challenges and opportunities in the advancement of nanomedicines. J Control Release. 2012; 164(2):236–246. 23064314 10.1016/j.jconrel.2012.10.007 PMC3504169

[18] Mignani S , Rodrigues J , Tomas H , Roy R , Shi X , Majoral J‐P. Bench‐to‐bedside translation of dendrimers: reality or utopia? A concise analysis. Adv. Drug Deliv. Rev 2017; 10.1016/j.addr.2017.11.007 29155170

[19] Sharma R , Kim S‐Y , Sharma A , et al. Activated microglia targeting dendrimer–minocycline conjugate as therapeutics for neuroinflammation. Bioconjugate Chem. 2017; 28(11):2874–2886. 10.1021/acs.bioconjchem.7b00569 PMC6023550 29028353

[20] Zhou Z , Ma X , Murphy CJ , et al. Molecularly precise dendrimer–drug conjugates with tunable drug release for cancer therapy. Angew Chem Int Ed. 2014; 53(41):10949–10955. 10.1002/anie.201406442 25155439

[21] Wang M , Liu H , Li L , Cheng Y. A fluorinated dendrimer achieves excellent gene transfection efficacy at extremely low nitrogen to phosphorus ratios. Nat Comms. 2014; 5:3053 10.1038/ncomms4053 24407172

[22] Sharma R , Zhang I , Shiao TC , Pavan GM , Maysinger D , Roy R. Low generation polyamine dendrimers bearing flexible tetraethylene glycol as nanocarriers for plasmids and siRNA. Nanoscale 2016; 8(9):5106–5119. 26868181 10.1039/c5nr06757j

[23] Lee CC , MacKay JA , Fréchet JMJ , Szoka FC. Designing dendrimers for biological applications. Nat Biotechnol. 2005;23(12):1517 16333296 10.1038/nbt1171

[24] Sharma A , Mejía D , Regnaud A , et al. Combined A3 coupling and click chemistry approach for the synthesis of dendrimer‐based biological tools. ACS Macro Lett. 2014; 3(10):1079–1083. 10.1021/mz5006298 35610796

[25] Sharma R , Zhang I , Abbassi L , Rej R , Maysinger D , Roy R. A fast track strategy toward highly functionalized dendrimers with different structural layers: an “onion peel approach”. Polym Chem. 2015; 6(9):1436–1444.

[26] Tomalia DA , Baker H , Dewald J , et al. A new class of polymers: Starburst‐Dendritic macromolecules. Polym J. 1985; 17(1):117–132.

[27] Kuruvilla SP , Tiruchinapally G , Crouch AC , ElSayed MEH , Greve JM. Dendrimer‐doxorubicin conjugates exhibit improved anticancer activity and reduce doxorubicin‐induced cardiotoxicity in a murine hepatocellular carcinoma model. PLoS ONE 2017; 12(8):1–24. 10.1371/journal.pone.0181944 PMC5567696 28829785

[28] Esfand R , Tomalia DA. Poly(amidoamine) (PAMAM) dendrimers: from biomimicry to drug delivery and biomedical applications. Drug Discov Today. 2001; 6(8):427–436. 11301287 10.1016/s1359-6446(01)01757-3

[29] Lesniak WG , Mishra MK , Jyoti A , et al. Biodistribution of fluorescently labeled PAMAM dendrimers in neonatal rabbits: effect of neuroinflammation. Mol Pharmaceutics. 2013;10(12):4560, 10.1021/mp400371r PMC3977004 24116950

[30] Kannan S , Dai H , Navath RS , et al. Dendrimer‐based postnatal therapy for neuroinflammation and cerebral palsy in a Rabbit model. Sci trans med. 2012; 4(130):130ra46–130ra46. 10.1126/scitranslmed.3003162 PMC3492056 22517883

[31] Nance E , Porambo M , Zhang F , et al. Systemic dendrimer‐drug treatment of ischemia‐induced neonatal white matter injury. J. Control. Release. 2015; 214:112–120. 26184052 10.1016/j.jconrel.2015.07.009 PMC4732874

[32] Mishra MK , Beaty CA , Lesniak WG , et al. Dendrimer brain uptake and targeted therapy for brain injury in a large animal model of hypothermic circulatory arrest. ACS Nano. 2014; 8(3):2134–2147. 24499315 10.1021/nn404872e PMC4004292

[33] Nemeth CL , Drummond GT , Mishra MK , et al. Uptake of dendrimer‐drug by different cell types in the hippocampus after hypoxic–ischemic insult in neonatal mice: Effects of injury, microglial activation and hypothermia. Nanomedicine 2017; 13(7):2359–2369. 28669854 10.1016/j.nano.2017.06.014 PMC5849407

[34] Iezzi R , Guru BR , Glybina IV , Mishra MK , Kennedy A , Kannan RM. Dendrimer‐based targeted intravitreal therapy for sustained attenuation of neuroinflammation in retinal degeneration. Biomaterials 2012; 33(3):979–988. 22048009 10.1016/j.biomaterials.2011.10.010

[35] Dai H , Navath RS , Balakrishnan B , et al. Intrinsic targeting of inflammatory cells in the brain by polyamidoamine dendrimers upon subarachnoid administration. Nanomedicine (London, England). 2010; 5(9):1317–1329. 10.2217/nnm.10.89 PMC3095441 21128716

[36] Guo Y , Johnson MA , Mehrabian Z , et al. Dendrimers target the ischemic lesion in rodent and primate models of Nonarteritic anterior ischemic optic neuropathy. PLOS ONE. 2016; 11(4):e0154437 27128315 10.1371/journal.pone.0154437 PMC4851377

[37] Lei J , Rosenzweig JM , Mishra MK , et al. Maternal dendrimer‐based therapy for inflammation‐induced preterm birth and perinatal brain injury. Sci Rep. 2017; 7(1):6106 28733619 10.1038/s41598-017-06113-2 PMC5522481

[38] Zhang F , Nance E , Zhang Z , et al. Surface functionality affects the biodistribution and microglia‐targeting of intra‐amniotically delivered dendrimers. J. Control. Release. 2016; 237:61–70. 27378700 10.1016/j.jconrel.2016.06.046 PMC5380001

[39] Nance E , Zhang F , Mishra MK , et al. Nanoscale effects in dendrimer‐mediated targeting of neuroinflammation. Biomaterials 2016; 101:96–107. 27267631 10.1016/j.biomaterials.2016.05.044 PMC5379995

[40] Jevprasesphant R , Penny J , Jalal R , Attwood D , McKeown NB , D'Emanuele A. The influence of surface modification on the cytotoxicity of PAMAM dendrimers. Int J Pharm. 2003; 252(1–2):263–266. 12550802 10.1016/s0378-5173(02)00623-3

[41] Thiagarajan G , Greish K , Ghandehari H. Charge affects the oral toxicity of poly(amidoamine) dendrimers. Eur J Pharm Biopharm. 2013; 84(2):330–334. 23419816 10.1016/j.ejpb.2013.01.019 PMC3860365

[42] Dobrovolskaia MA , Patri AK , Simak J , et al. Nanoparticle size and surface charge determine effects of PAMAM dendrimers on human platelets in vitro. Mol Pharmaceutics. 2012; 9(3):382–393. 10.1021/mp200463e PMC3624701 22026635

[43] Brok J , Buckley N , Gluud C. Interventions for paracetamol (acetaminophen) overdoses. Cochrane Database Syst Rev. 2002; (3): 10.1002/14651858.CD003328 12137690

[44] Wang X , Svedin P , Nie C , et al. N‐acetylcysteine reduces lipopolysaccharide‐sensitized hypoxic‐ischemic brain injury. Ann Neurol. 2007; 61(3):263–271. 17253623 10.1002/ana.21066

[45] Olsson B , Johansson M , Gabrielsson J , Bolme P. Pharmacokinetics and bioavailability of reduced and oxidized N‐acetylcysteine. Eur J Clin Pharmacol. 1988; 34(1):77–82. 3360052 10.1007/BF01061422

[46] Olney J , Zorumski C , Price M , Labruyere J. L‐cysteine, a bicarbonate‐sensitive endogenous excitotoxin. Science 1990; 248(4955):596–599. 2185543 10.1126/science.2185543

[47] Kambhampati SP , Mishra MK , Mastorakos P , Oh Y , Lutty GA , Kannan RM. Intracellular delivery of dendrimer triamcinolone acetonide conjugates into microglial and human retinal pigment epithelial cells. Eur J Pharm Biopharm. 2015; 95:239–249. 25701805 10.1016/j.ejpb.2015.02.013 PMC4861086

[48] Wang B , Navath RS , Romero R , Kannan S , Kannan R. Anti‐inflammatory and anti‐oxidant activity of anionic dendrimer – N‐acetyl cysteine conjugates in activated microglial cells. Int J Pharm. 2009; 377(1–2):159–168. 19463931 10.1016/j.ijpharm.2009.04.050 PMC3917717

[49] Zhang Z , Bassam B , Thomas AG , et al. Maternal inflammation leads to impaired glutamate homeostasis and up‐regulation of glutamate carboxypeptidase II in activated microglia in the fetal/newborn rabbit brain. Neurobiol Dis. 2016; 94:116–128. 27326668 10.1016/j.nbd.2016.06.010 PMC5394739

[50] Williams M , Zhang Z , Nance E , et al. Maternal inflammation results in altered tryptophan metabolism in Rabbit Placenta and fetal brain. Dev Neurosci. 2017; 39(5):399–412. 28490020 10.1159/000471509 PMC6447288

[51] Zhang Z , Jyoti A , Balakrishnan B , et al. Trajectory of inflammatory and microglial activation markers in the postnatal rabbit brain following intrauterine endotoxin exposure. Neurobiol Dis. 2018; 111:153–162. 29274431 10.1016/j.nbd.2017.12.013 PMC6082145

[52] Nance E , Kambhampati SP , Smith ES , et al. Dendrimer‐mediated delivery of N‐acetyl cysteine to microglia in a mouse model of Rett syndrome. J Neuroinflammation. 2017; 14(1):252 29258545 10.1186/s12974-017-1004-5 PMC5735803

[53] Perumal OP , Inapagolla R , Kannan S , Kannan RM. The effect of surface functionality on cellular trafficking of dendrimers. Biomaterials 2008; 29(24–25):3469–3476. 18501424 10.1016/j.biomaterials.2008.04.038

[54] Smith SM , Wunder MB , Norris DA , Shellman YG. A simple protocol for using a LDH‐based cytotoxicity assay to assess the effects of death and growth inhibition at the same time. PLOS ONE. 2011; 6(11):e26908 22125603 10.1371/journal.pone.0026908 PMC3219643

[55] Kannan G , Kambhampati SP , Kudchadkar SR. Effect of anesthetics on microglial activation and nanoparticle uptake: Implications for drug delivery in traumatic brain injury. J. Control. Release. 2017; 263:192–199. 28336376 10.1016/j.jconrel.2017.03.032

[56] Sharma R , Kottari N , Chabre YM , Abbassi L , Shiao TC , Roy R. A highly versatile convergent/divergent “onion peel” synthetic strategy toward potent multivalent glycodendrimers. Chem. Commun. 2014; 50(87):13300–13303. 10.1039/c4cc06191h 25227948

[57] Killops KL , Campos LM , Hawker CJ. Robust, efficient, and orthogonal synthesis of dendrimers via thiol‐ene “click” chemistry. J Am Chem Soc. 2008; 130(15):5062–5064. 18355008 10.1021/ja8006325

