# Supplementary material for: Scalable synthesis and validation of PAMAM dendrimer‐N‐acetyl cysteine conjugate for potential translation
Source: Bioeng Transl Med. 2018 May 25;3(2):87–101. doi: 10.1002/btm2.10094 (PMC6063872; doi:10.1002/btm2.10094)
Supplement: Supplementary file 1 — Supporting Information [file BTM2-3-87-s001.docx]

**ELECTRONIC SUPPORTING INFORMATION**

**Scalable synthesis and validation of PAMAM dendrimer-*N*-acetyl cysteine conjugate for potential translation**

Rishi Sharma^1^, Anjali Sharma^1^, Siva P. Kambhampati^1^, Rajsekar Rami Reddy^1^, Zhi Zhang^2^, Jeffrey L. Cleland^3^, Sujatha Kannan^1,2,4,5^, and Rangaramanujam M. Kannan^*1,4,5,6^

^1^Center for Nanomedicine, Department of Ophthalmology, Wilmer Eye Institute Johns Hopkins University School of Medicine, Baltimore, MD 21231, USA;

^2^*Department of Anesthesiology and Critical Care Medicine, Johns Hopkins University School of Medicine, Baltimore, MD 21287, USA;*

^3^*Ashvattha Therapeutics, LLC, 855 North Wolfe Street, Baltimore, MD 21218,* USA;

^4^*Hugo W. Moser Research Institute at Kennedy Krieger, Inc., Baltimore MD, 21205,* USA;

^5^*Kennedy Krieger Institute – Johns Hopkins University for Cerebral Palsy Research Excellence, Baltimore, MD 21218,* USA;

^6^*Department of Chemical and Biomolecular Engineering, Johns Hopkins University, Baltimore MD, 21218,* USA;

^*^Corresponding author:

Rangaramanujam M. Kannan, Arnall Patz Distinguished Professor of Ophthalmology, Center for Nanomedicine at the Wilmer Eye Institute, 400 North Broadway, Baltimore, Maryland 21231, USA

Tel.: +1 443-287-8634; Fax: +1 443-287-8635; e-mail: krangar1@jhmi.edu

Contents

1. Synthesis and characterization of intermediates and dendrimers
2. Characterization data of intermediates and dendrimer conjugates
3. **Synthesis and characterization of intermediates and dendrimers**

All reactions in the organic medium were performed in standard oven-dried glassware under an inert nitrogen atmosphere using anhydrous solvents. All the solvents were deoxygenated before use when necessary by purging with nitrogen. Dialysis membrane was purchased from Spectrum Laboratories Inc. Ethylenediamine-core poly(amidoamine) (PAMAM) dendrimer (Pharma grade generation 4 consisting 64 hydroxyl end-groups) (G4-OH) was purchased from Dendritech Inc. Prior to use, methanol was removed under reduced pressure to yield a viscous oil. Dendrimer was dissolved in HPLC grade water, lyophilized and dried to yield hygroscopic white sticky material. Dendrimer was stored at -20^0^C under argon atmosphere. All reagents were used as supplied without prior purification unless otherwise stated and obtained from Sigma-Aldrich Chemical Co. Ltd. Cy5-mono-NHS ester was purchased from Amersham Biosciences-GE Healthcare. Lipopolysaccharide (LPS) was purchased from Sigma. Reactions were monitored by analytical thin-layer chromatography (TLC) using silica gel 60 F254 precoated plates (E. Merck) and compounds were visualized by 254 nm light, KMnO_4_ staining solution and subsequent development by gentle warming with a heat-gun. Purifications were performed by combi-flash column chromatography system from Biotage using prepacked silica gel column with the indicated eluent.

**1.1 Preparation of compound 7:**

To a stirred solution of PAMAM G4-OH **1** (10.0 g, 0.70 mmoles, **compound 1**) in anhydrous *N*, *N* dimethyl formamide (DMF, 100 ml) added BOC-GABA-OH (5.124 g, 25.212 mmoles), DMAP (3.42 g, 28.013 mmoles) and stirred at RT for 5 minutes to make a clear solution. EDC.HCl (6.04 g, 31.500 mmoles) was added in portions to the reaction mixture over the period of 5 minutes. The reaction mixture was stirred for 36 h at room temperature. The reaction mixture was transferred to 1kD MW cut-off cellulose dialysis tubing and dialysed against water for 24 h, periodically changing water 4-5 times. The contents of dialysis tubing were transferred to pre-weighed 50 mL falcon tube and lyophilized to get desired product, **compound 7** as white fluffy hygroscopic solid. Yield: 85%, 10.88 g.

^1^H NMR (500 MHz, DMSO) δ 8.07 (m, 124 internal amide H), 6.82 (s, Gaba amide H), 4.70 (s, surface OH, 22H), 3.99 (s, ester linked 46H), 3.39 (d, *J* = 5.0 Hz, dendrimer -CH_2_), 3.27 (d, *J* = 5.3 Hz, dendrimer-CH_2_), 3.19 – 3.02 (m, dendrimer-CH_2_), 2.92 (d, *J* = 6.2 Hz, dendrimer-CH_2_), 2.70 - 2.55 (m, dendrimer-CH_2_ ), 2.45 - 2.39 (m, dendrimer-CH_2_), 2.23 – 2.12 (m, dendrimer-CH_2_), 1.67 – 1.56 (m, Gaba linker-CH_2_, 48H), 1.36 (s, BOC- 220H). Compound 2 is >97 % pure, Retention time: 26 min.

**1.2 Preparation of compound 8:**

BOC protected dendrimer **2** (10 g, 0.545mmoles) was placed in a flame dried 250 ml round bottom flask and 80 ml of dichloromethane (DCM) was added to the compound under Nitrogen atmosphere. The reaction mixture was sonicated for 15 minutes to make a cloudy suspension followed by the addition of 20 mL of TFA dropwise while stirring at 0^o^C. The solution turned clear with the addition of TFA but became very thick with time. Reaction mixture was stirred vigorously for 12 h at room temperature. The color of the reaction changed from colorless to light brownish color. Once completed, DCM was evaporated. The reaction mixture was diluted with methanol and evaporated using rotary evaporator. This procedure was repeated until excess of TFA was completely removed. The reaction mixture was left at high vacuum for 3 h to remove any trace of solvents to afford compound **8** as an off-white fluffy hygroscopic material, which was directly used for the next step without any further purification.

^1^H NMR (500 MHz, DMSO) δ 8.61-7.91 (m, 145H), 4.42 – 4.38 (m, surface-OH), 4.08 – 3.94 (m, ester linked H), 3.63 – 3.24 (m, dendrimer -CH_2_), 3.22 – 3.07 (m, dendrimer -CH_2_), 2.87 – 2.79 (m, dendrimer -CH_2_), 2.70 – 2.56 (m, dendrimer -CH_2_), 2.42 (t, *J* = 7.2 Hz, 46H), 1.93 – 1.59 (m, Gaba linker-CH_2_). HPLC Purity: >96 %, Retention time: 19.2 min

**1.3 Preparation of compound 9 (*N*-acetyl-S-((3-((2,5-dioxopyrrolidin-1-yl)oxy)-3-oxopropyl)thio)cysteine [SPDP-NAC linker]):**

A flame-dried 100 mL round bottom flask was charged with *N*-succinimidyl 3-(2-pyridyldithio)-propionate (SPDP) (10 g, 32.04 mmoles) followed by the addition of anhydrous tetrahydrofuran (THF, 30 mL) under inert atmosphere with constant stirring. A drop-wise addition of a solution of *N*-acetyl cysteine (NAC, 5.74 g, 35.24 mmoles, 1.1 eq) dissolved in THF (30 mL) was performed. The reaction mixture turned yellow within few minutes. The reaction mixture was stirred at RT for 4 h. Reaction was monitored by TLC and once the starting material (SPDP) was consumed, the solvent was removed using rotary evaporator. The crude product was purified using pre-packaged high performance redisep gold Rf™ 80-gram silica cartridge on CombiFlash system from Teledyne keeping the flow 60 mL/minute. The column was started in DCM and the pure desired product was collected in 4% MeOH in dichloromethane as white powder in 75.4% yield (8.8 g).

^1^H NMR (500 MHz, CDCl_3_): δ 6.67 (d, *J* = 6.9 Hz, 1H), 4.87 (d, *J* = 5.5 Hz, 1H), 3.31 (ddd, *J* = 48.8, 14.2, 4.7 Hz, 2H), 3.13 – 2.99 (m, 4H), 2.88 (s, 4H), 2.09 (s, 3H).^13^C NMR (126 MHz, CDCl_3_) δ 171.9, 171.2, 167.0, 52.0, 40.0, 32.8, 31.1, 25.5, 22.9. **HRMS (ESI^+^)** *m/z* calc. C_12_H_16_N_2_O_7_S_2_is 364.39, Found: 387.02 {M+Na^+^}. HPLC purity: > 99 %, Retention time 25.9 min

**1.4 Preparation of compound 10 (D-NAC)**:

A flame dried 500 mL round bottom flask was charged with compound **8** (12g) followed by the addition of anhydrous DMF (80 mL) under inert atmosphere with constant stirring. The flask was sonicated and vortexed until it made a clear solution. The pH of the reaction mixture was adjusted to 7.0 - 7.5 by addition of *N*, *N* diisopropylethylamine (DIPEA). The reaction mixture was stirred for 30 minutes and once the pH was stable, slow addition of compound **9** (7.34 g, 20.1 mmoles, 27eq) dissolved in DMF (40 mL) was performed. The reaction mixture was stirred under nitrogen at room temperature for 12 h. It was then transferred to 1000 cut-off dialysis bag and dialysed against DMF for 6 h followed by water for 24 h, periodically changing solvent every 2-3 h. The contents of dialysis tubing were transferred to pre-weighed 50 mL falcon tubes and were lyophilized to get Dendrimer-*NAC* conjugate **10** as white solid. Yield: 90%, 14.0 g. The extent of final conjugation was calculated comparing N*H* protons of dendrimer in between 8-7.5 ppm to *N*-acetyl protons of NAC at 1.8 ppm and –C*H* proton of NAC around 4.4 ppm.

^1^H NMR (500 MHz, DMSO) δ 8.30 – 7.75 (m, amide-170H), 4.41 (s, NAC-CH-23H), 4.00 (s, ester linked-46H), 3.43 – 3.34 (m, , dendrimer -CH_2_), 3.18 – 3.03 (m, , dendrimer -CH_2_), 2.94 – 2.82 (m, dendrimer -CH_2_), 2.75 – 2.65 (m, , dendrimer -CH_2_), 2.35 – 2.13 (m, , dendrimer -CH_2_), 1.86 (s, *N-*Acetyl, 72H), 1.64 (s, GABA linker-CH_2_-46H). HPLC purity: > 96 %, retention time: 22.4 min

**1.5 Preparation of compound 11 (D-Allyl)**:

To a stirred solution of PAMAM-G4-OH (**1**, 530mg, 0.037mmoles) in dry DMF (15mL), NaH (200mg, 8.33mmoles) was added in portions at 0^o^C. After 15 minutes, allyl bromide (0.127mL, 1.48mmoles) was added and the stirring was continued for 24h at room temperature. The solution was then dialyzed against DMF followed by water for 24h. The aqueous solution was lyophilized to get product **11** as white powder in 45% yield.

^1^H NMR (500 MHz, DMSO) δ 8.20-7.70 (m, 124 internal amide H), 5.89-5.83 (m, allyl -CH), 5.26-5.12 (dd, allyl –CH_2_), 3.92 (d, allyl –CH_2_), 3.39 (d, *J* = 5.0 Hz, dendrimer -CH_2_), 3.40-3.37 (m, dendrimer-CH_2_), 3.19 – 3.21 (m, dendrimer-CH_2_), 3.12-3.10 (m, dendrimer-CH_2_), 2.74 - 2.70 (m, dendrimer-CH_2_ ), 2.51 - 2.45 (m, dendrimer-CH_2_), 2.23 – 2.12 (m, dendrimer-CH_2_).

**1.6 Preparation of compound 12**:

To a stirred solution of compound **11** (192mg, 0.012mmoles) in DMF (5mL), 2-(boc-amino) ethanethiol (200mg, 1.12mmoles) was added followed by the addition of catalytic amount of [2,2-dimethoxy-2-phenylacetophenone](http://www.sigmaaldrich.com/catalog/product/aldrich/196118) (DMPAP). The reaction mixture was stirred under UV light (365 nm) for 24h. The reaction was dialyzed against DMF followed by water for 24h. The aqueous solution was lyophilized to get product **12** as white powder in 78% yield.

^1^H NMR (500 MHz, DMSO) δ 7.10-6.90 (m, 124 internal amide H), 6.57 (m, NH) 3.6-3.18 (m, dendrimer -CH_2_), 3.12-3.10 (m, dendrimer-CH_2_), 2.76 - 2.70 (m, dendrimer-CH_2_ ), 2.54 - 2.45 (m, dendrimer-CH_2_), 2.20 – 2.12 (m, dendrimer-CH_2_), 1.86 (t, linker –CH_2_), 1.72 (t, linker –CH_2_), and 1.38 (bs, BOC H).

**1.7 Preparation of compound 13**:

To a stirred solution of compound **12** (200mg) in DCM (6mL), trifluoroacetic acid (2mL) was added and stirring was continued for 4 h. The solvent was evaporated under reduced pressure. Methanol was added and evaporated several times to remove TFA. The residue was dried under reduced pressure to afford hygroscopic solid **13** in quantitative yield.

^1^H NMR (500 MHz, DMSO) δ 8.70-7.90 (m, 124 internal amide H), 3.60-3.25 (m, dendrimer-CH_2_), 3.23 – 3.05 (m, dendrimer-CH_2_), 3.05-2.70 (m, dendrimer-CH_2_), 2.69 - 2.56 (m, dendrimer-CH_2_ and linker –CH_2_ ), 2.51 - 2.45 (m, dendrimer-CH_2_), 2.23 – 2.12 (m, dendrimer-CH_2_), 1.86 (t, linker –CH_2_), and 1.72 (t, linker –CH_2_).

**1.8 Preparation of compound 14 (D-NAC^a^ via ether linker)**:

A flame dried 500 mL round bottom flask was charged with compound **13** (300mg, 0.018mmoles) followed by the addition of anhydrous DMF (10mL) under inert atmosphere with constant stirring. The flask was sonicated and vortexed until it made a clear solution. The pH of the reaction mixture was adjusted to 7.0 - 7.5 by addition of DIPEA. The reaction mixture was stirred for 30 minutes and once the pH was stable, slow addition of compound **9** (331mg, 0.909mmoles) dissolved in DMF (10 mL) was performed. The reaction mixture was stirred under nitrogen at room temperature for 12 h. It was then transferred to 1000 cut-off dialysis bag and dialysed against DMF for 6 h followed by water for 24 h, periodically changing solvent every 2-3 h. The contents of dialysis tubing were transferred to pre-weighed 50 mL falcon tubes and lyophilized to get D-NAC conjugate **14** as white solid in 62% yield.

^1^H NMR (500 MHz, DMSO) δ 8.40 – 7.80 (m, amide H), 4.32 (s, NAC-CH), 3.50 – 3.34 (m, dendrimer -CH_2_), 3.25 – 3.10 (m, , dendrimer -CH_2_), 2.94 – 2.82 (m, dendrimer -CH_2_), 2.78 – 2.65 (m, dendrimer -CH_2_), 2.52 – 2.36 (m, , dendrimer -CH_2_ and linker –CH_2_), 2.20 (m, linker –CH_2_), 1.84 (s, *N-*Acetyl, 72H), 1.72 (s, linker-CH_2_).

- 1. **Preparation of compound 15 (Cy5-D-NAC)**:

A flame dried 500 mL round bottom flask was charged with compound **8** (200mg) followed by the addition of anhydrous DMF (10 mL) under inert atmosphere with constant stirring. The flask was sonicated and vortexed until it made a clear solution. The pH of the reaction mixture was adjusted to 7.0 - 7.5 by addition of DIPEA. The reaction mixture was stirred for 30 minutes and once the pH was stable, addition of Cy5-NHS ester (1.2eq) was done and the reaction was stirred for 2 h. This was followed by the addition of compound **9** (25eq) dissolved in DMF (5mL). The reaction mixture was stirred under nitrogen at room temperature for 12 h. It was then transferred to 1000 cut-off dialysis bag and dialysed against DMF for 6 h followed by water for 24 h, periodically changing solvent every 2-3 h. The contents of dialysis tubing were transferred to pre-weighed 50 mL falcon tubes and were lyophilized to get Cy5-D-NAC conjugate **15** as blue solid. Yield: 80%.

^1^H NMR (500 MHz, DMSO) δ 8.33 – 7.75 (m, internal amide H), 7.66 (s, Cy5 H), 7.32 (s, Cy5 H), 7.01 (s, Cy5 H), 6.58 (s, Cy5 H), 6.31 (s, Cy5 H), 4.49 – 4.30 (m, NAC -CH), 4.00 (m, ester -CH_2_), 3.39 (m, dendrimer H), 3.27 (m, dendrimer H), 3.20 – 2.99 (m, dendrimer H), 2.89 (m, dendrimer H), 2.71 (m, dendrimer H), 2.37 – 2.12 (m, dendrimer H), 1.86 (s, NAC -CH_3_), 1.72 – 1.55 (m, GABA -CH_2_), 1.25 (Cy5 H).

**2. Characterization of intermediates and dendrimers**

**Compound 7**

**Figure S1.** ^1^H NMR of Compound **7** in in DMSO-d6.


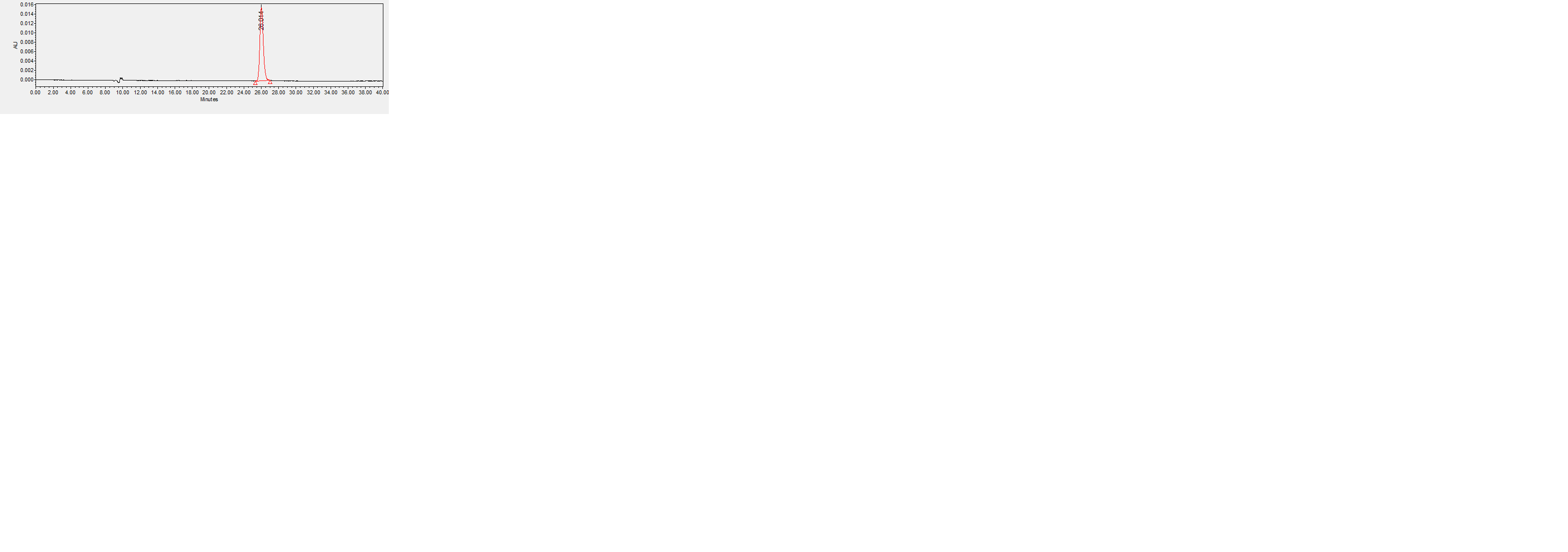


**Figure S2.** HPLC chromatogram of Compound **7** monitored at 210 nm wavelength

**Compound 8**

**Figure S3.** ^1^H NMR of Compound **8** in DMSO-d6.


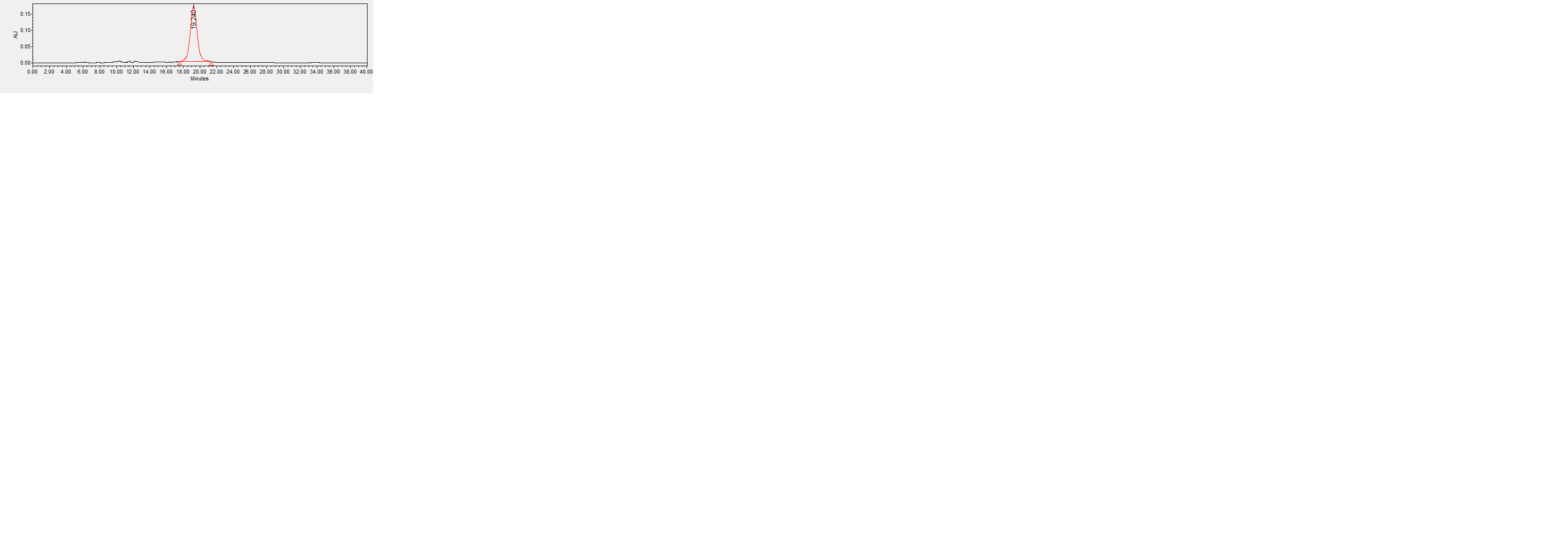


**Figure S4.** HPLC chromatogram of Compound **8** monitored at 210 nm wavelength

**Compound 9**

**Figure S5.** ^1^H NMR (500 MHz, CDCl_3_) of Compound **9**

**Figure S6.** ^13^C NMR of Compound **9**


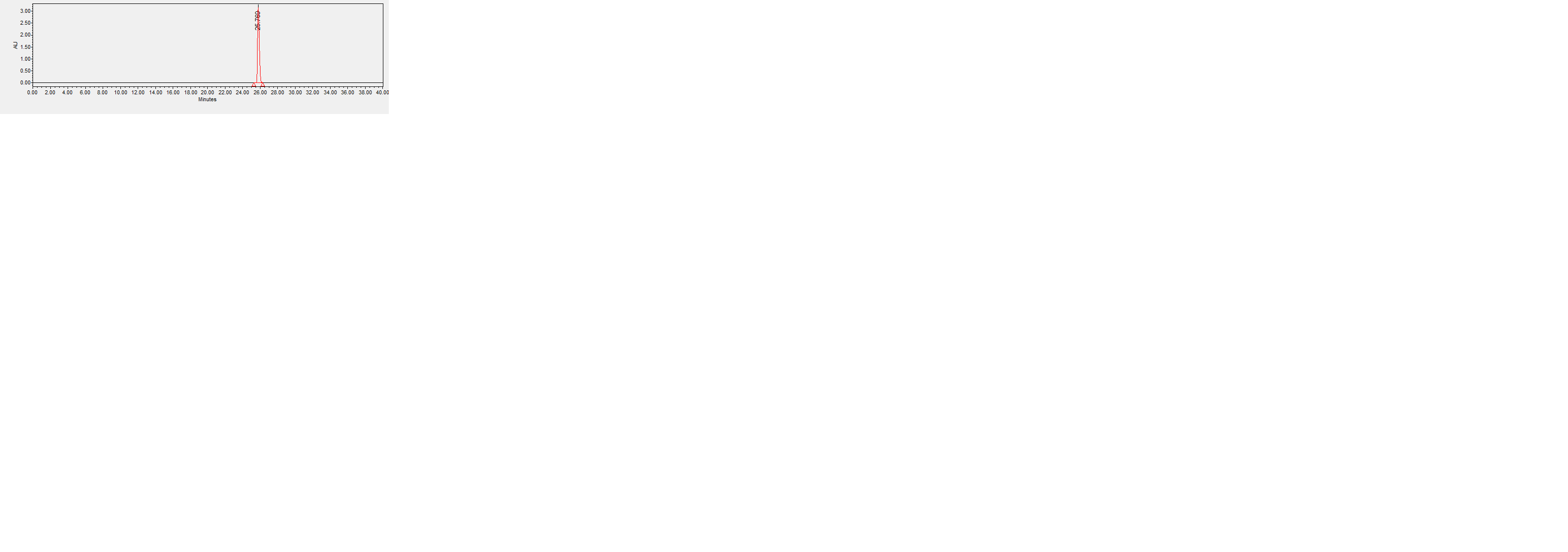


**Figure S7.** HPLC chromatogram of Compound **9**

**Figure S8.** HRMS of the of Compound **9**

**Compound 10 (D-NAC)**

**Figure S9.** ^1^H NMR of Dendrimer-NAC conjugate **10** in DMSO**.**

**Figure S10.** ^13^C NMR of Dendrimer-NAC conjugate **10** in DMSO**.**


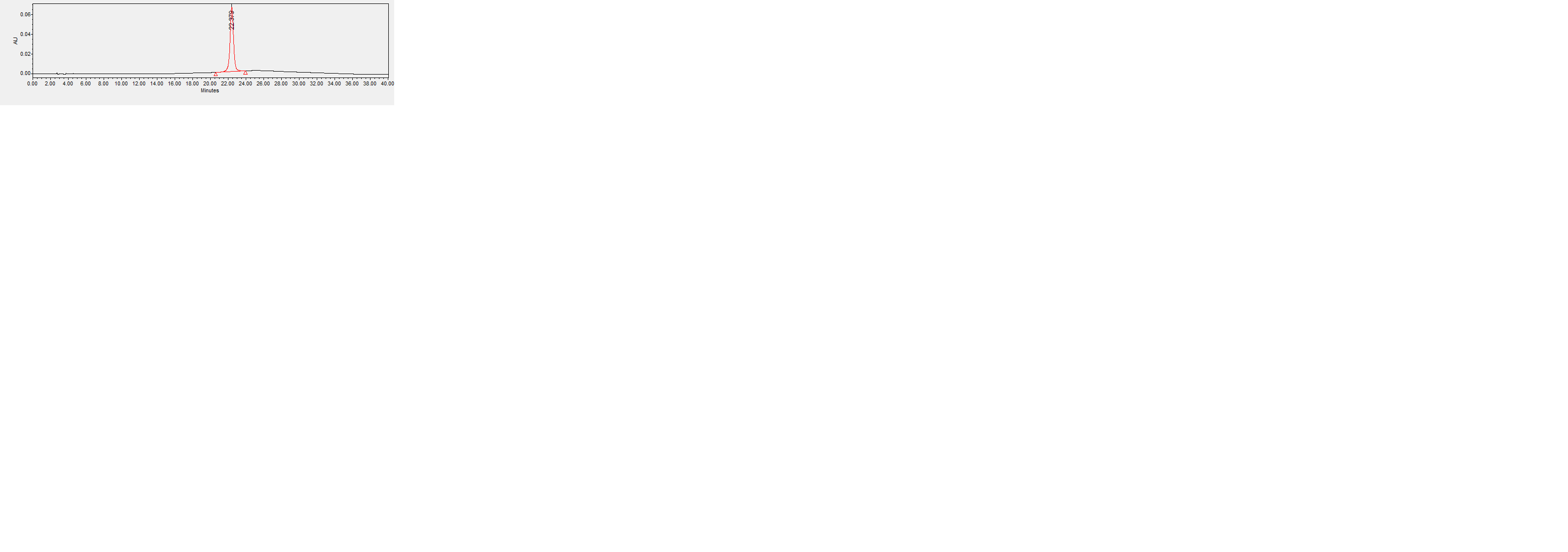


**Figure S11.** HPLC chromatogram of D-NAC (**10**) monitored at 210 nm wavelength

**Compound 11**

**Figure S12.** ^1^H NMR of compound **11** in DMSO**.**

**Compound 12**

**Figure S13.** ^1^H NMR of compound **12** in DMSO**.**

**Compound 13**

**Figure S14.** ^1^H NMR of compound **13** in DMSO**.**

**Compound 14 (D-NAC^a^ via ether linker)**

**Figure S15.** ^1^H NMR of dendrimer-NAC conjugate via ether linker (**D-NAC^a^, 14**) in DMSO**.**

**
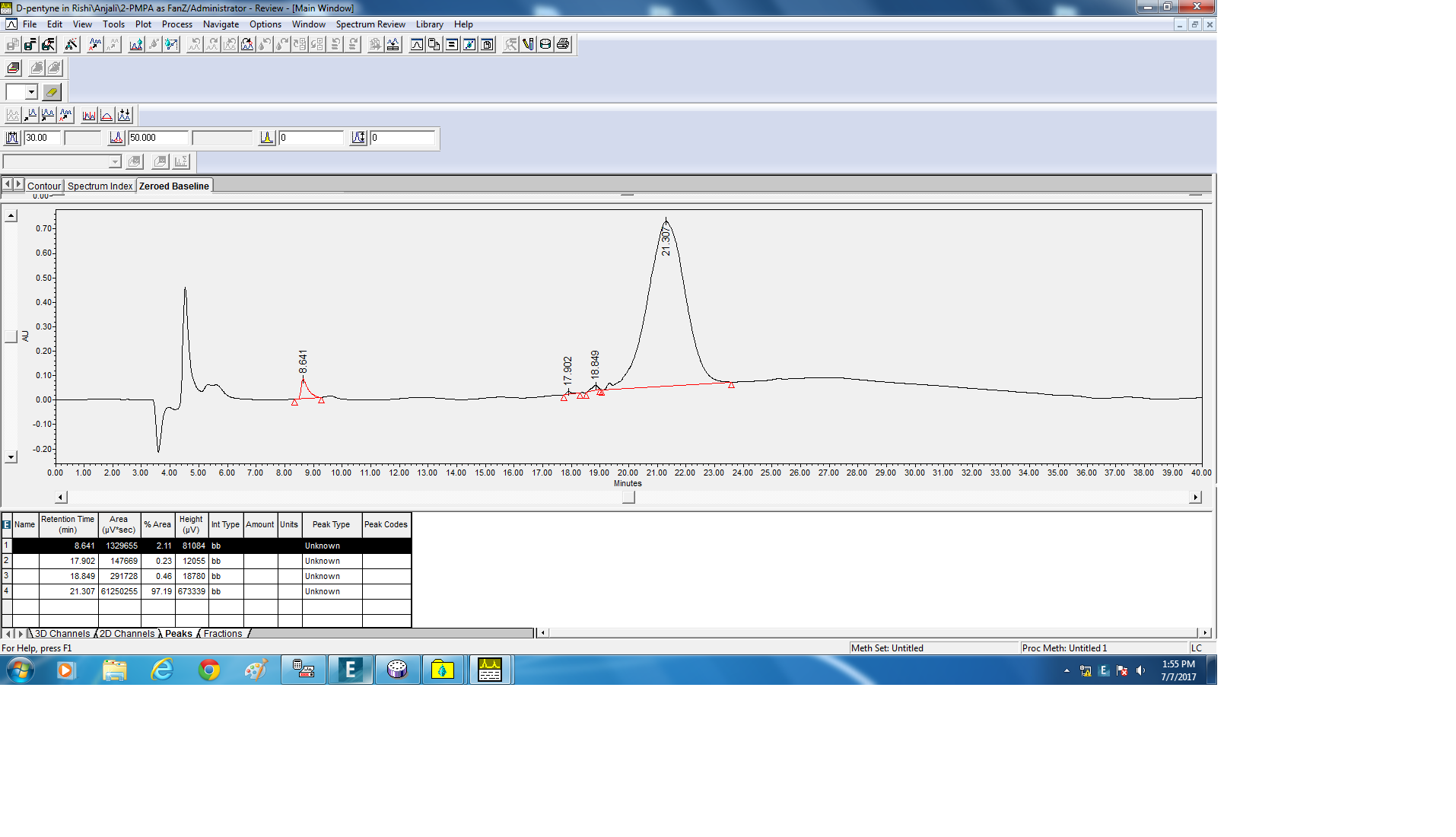
**

**Figure S16.** HPLC chromatogram of dendrimer-NAC conjugate via ether linker (**D-NAC^a^, 14**).

**Compound 15 (Cy5-D-NAC)**

**Figure S19.** ^1^H NMR of Cy5-D-NAC conjugate (**15**) in DMSO**.**

**
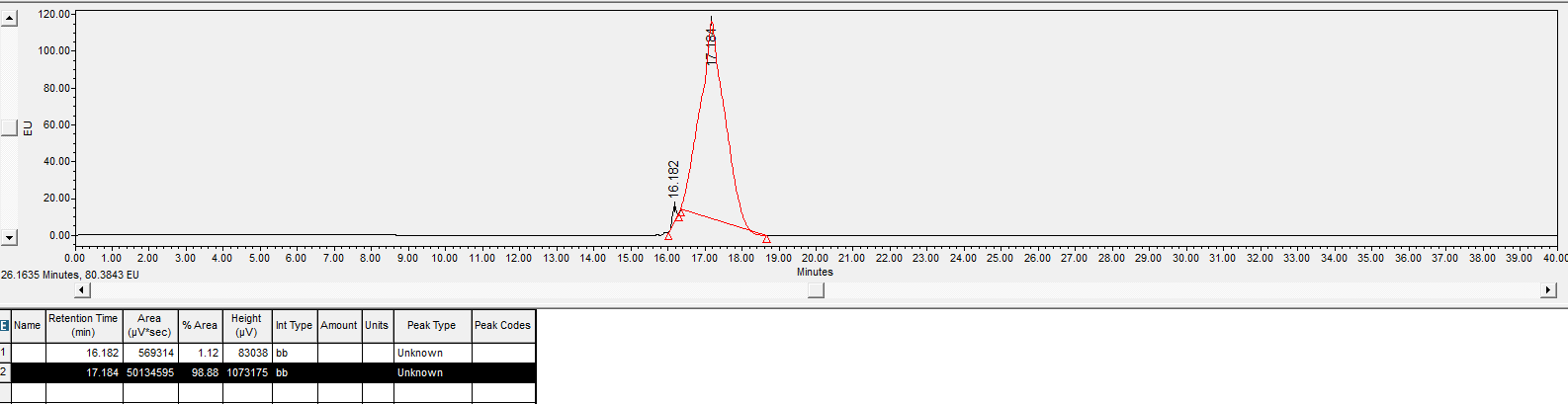
**

**Figure S20.** HPLC chromatogram of Cy5-D-NAC conjugate (**15**) at 650 nm**.**
